# Visualising ionic screening in perovskite solar cells: a bumpy ride along the *J*–*V* curve

**DOI:** 10.1039/d5el00133a

**Published:** 2025-08-27

**Authors:** Miguel A. Torre Cachafeiro, Stéphanie Narbey, Beat Ruhstaller, Frank Nüesch, Wolfgang Tress

**Affiliations:** a Institute of Computational Physics, Zurich University of Applied Sciences (ZHAW) 8400 Winterthur Switzerland miguel.torre@zhaw.ch wolfgang.tress@zhaw.ch; b Institut des Matériaux, École Polytechnique Fédérale de Lausanne (EPFL) 1015 Lausanne Switzerland; c Solaronix SA 1170 Aubonne Switzerland; d Fluxim AG 8400 Winterthur Switzerland; e Laboratory for Functional Polymers, Swiss Federal Laboratories for Materials Science and Technology (Empa) 8600 Dübendorf Switzerland

## Abstract

The current density–voltage (*J*–*V*) curve of perovskite solar cells (PSCs) commonly depends on the voltage scanning rate and direction, due to the presence of mobile ionic charges which screen the electric field, lowering the total driving force for charge extraction. In this study, experimental data and drift-diffusion simulations are combined to provide new insights into scan rate dependent *J*–*V* curves, focusing on triple mesoscopic carbon-based PSCs (CPSCs), which show a distinct current overshoot (‘bump’) in the backward scan which had not been fully explained until now. Additionally, the thickness optimisation problem in CPSCs is shown to be governed by the ionic distribution, which determines the ability to collect charge photogenerated in the ZrO_2_ layer. Using simulations, we provide intuitive visual representations of the changes in electric field across the perovskite absorber during voltage scans at different rates, which determine the hysteresis and occurrence of the bump as a result of the polarity inversion of ionic space charge layers. The spatial maps obtained are directly correlated with experimental temperature- and voltage-dependent measurements of external quantum efficiency (EQE), offering an innovative and effective method to visualise ionic screening. This study introduces significant insights for the design and optimisation of CPSC devices considering ionic effects and presents a versatile characterisation approach applicable to all PSC architectures.

## Abstract 2

The *J*–*V* bump and hysteresis in perovskite solar cells are studied combining measurement and simulation, linking them to spatial collection efficiency changes from ionic charge accumulation, as visualised in external quantum efficiency (EQE) spectra.

## Graphical abstract

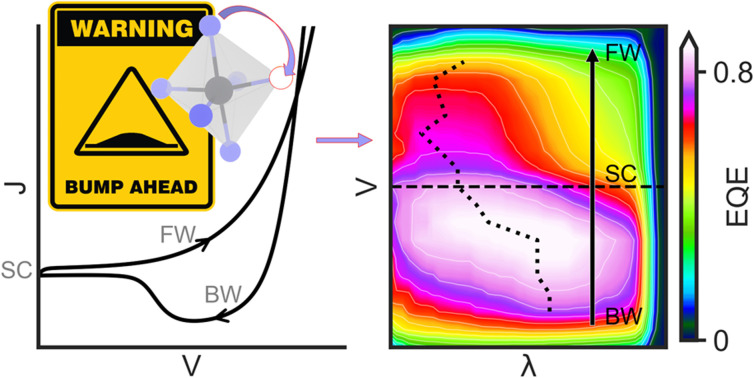

**Broader context**

In recent years, lead halide perovskite-based solar cells (PSCs) have emerged as a potentially disruptive photovoltaic technology, offering high power conversion efficiencies (PCEs) with low manufacturing costs and simple fabrication *via* solution processing. Perovskite semiconductors are quite unique; despite their mixed ionic-electronic conductivity and the high defect densities expected from solution processing, PSCs can easily achieve high PCE values. While the defects most likely to form mostly induce shallow energy levels close to the band gap edges, ionic defects are mobile and often critically affect the performance of PSCs as they redistribute to screen the electric field. The effects of mobile ions on the transient *J*–*V* response of PSCs are investigated through a combined experimental and simulation approach, focusing specifically on a characteristic current maximum, or bump, which is often observed. Fully-printable carbon-based PSCs (CPSCs) are used as an example, since the bump is highly common for such architectures, which have emerged as a solution towards higher stability using abundant materials. The findings shed light on the device physics of PSCs and provide new characterization approaches useful towards device optimisation.

## 1 Introduction

Lead halide perovskite solar cells (PSCs) have rapidly achieved over 25% solar radiation to electrical power conversion efficiency (PCE),^1^ showing their potential as a game-changer in photovoltaic technology. Current density against applied voltage (*J*–*V*) curves under illumination provide the most straightforward method for identifying performance metrics of a solar cell, such as the PCE. The *J*–*V* curve of PSCs often shows a scan rate-dependent hysteresis,^2^ mainly due to the mixed ionic-electronic conductivity of these materials.^3,4^ Hysteresis can emerge at intermediate scan rates, where the speed is neither too fast for ionic response to occur nor slow enough for mobile ions to fully track changes in the applied voltage.^5^ When scanning the voltage in the backward direction, *i.e.* decreasing the voltage from a high forward bias precondition, a higher short circuit current density (*J*_SC_) is often seen for higher scan rates, with lower scan rates resulting in lower *J*_SC_; this *J*_SC_ loss aggravates with aging^2^ as it is caused by ion-induced current collection losses which seem to depend crucially on the density of mobile ionic charges.^6,7^

In addition to these effects, a current overshoot (‘bump’) in *J*–*V* backward scan curves has been observed in multiple studies across different device architectures.^8–13^ This is an intriguing feature as lowering the voltage reduces photocurrent, something that does not commonly happen in standard solar cells. For mesoscopic carbon-based PSCs (CPSCs), the occurrence of the bump and *J*_SC_ difference has been characterised in detail by De Moor *et al.*,^11^ where this phenomenon was attributed to a temperature-activated ionic effect, in agreement to numerous reports linking hysteresis to ion migration (iodine vacancies) in lead halide perovskites.^14^ However, a better physical understanding of ion redistribution as a likely cause of a bump in the *J*–*V* curve requires further systematic investigations, especially to clarify why *J*–*V* hysteresis may manifest with and without bump. In this study, we take a closer look at the underlying mechanisms behind scan rate-dependent current collection losses and the characteristic bump observed in the *J*–*V* curves of CPSCs.

**Fig. 1:**
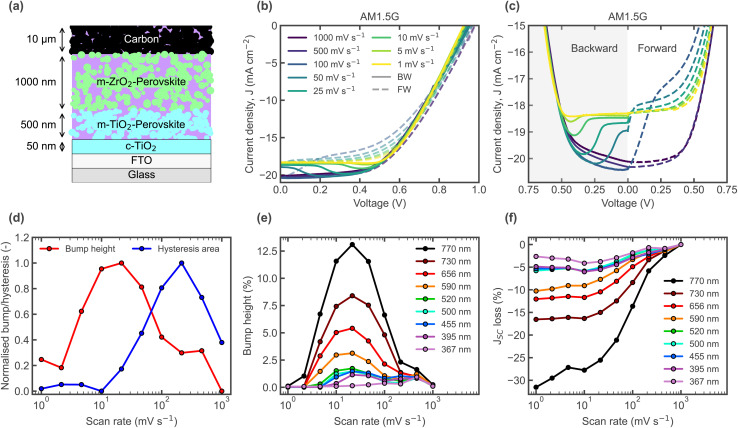
(a) CPSC device stack schematic. (b) *J*–*V* curves measured under solar simulator illumination, with varying scan rates, starting with the backward scan from a precondition at *V*_OC_. (c) Same data as in (b) but focusing on the *J*_SC_ level. (d) Hysteresis area and relative bump height (normalised) for varying scan rates, computed from *J*–*V* curves measured under white LED illumination. (e) Relative bump heights and (f) relative *J*_SC_ loss (with respect to the *J*_SC_ of the fastest scan rate), from *J*–*V* curves taken with monochromatic LEDs of various wavelengths at comparable photon flux.

PSCs generally consist of a perovskite absorber between selective layers, also known as charge transport layers (CTLs). PSCs can be realised with different architectures; using fully planar stacks in an n–i–p or p–i–n order, introducing a mesoporous electron transport layer (ETL), or even in a fully mesoscopic configuration without a hole transport layer (HTL). The latter, including a carbon-based electrode (CPSCs), have emerged as a promising solution to address the stability and scalability challenges faced by other architectures.^15–18^ However, their performance in terms of PCE still lags behind the record efficiencies obtained with other single-junction PSC stacks. In CPSCs, perovskite is infiltrated as a last fabrication step onto the mesoporous scaffold comprised of titania (*m*-TiO_2_)/zirconia (*m*-ZrO_2_)/carbon deposited on a compact layer of TiO_2_ on top of fluorine-doped tin oxide (FTO) glass, as depicted in Fig. 1a. The TiO_2_ layer is the ETL, whereas the ZrO_2_ layer acts mainly as a spacer and no HTL is used. A comprehensive summary of this device architecture is given by Han *et al.*^19^ The fabrication details for the CPSCs devices used in this study can be found in the SI. The perovskite consists of methylammonium lead triiodide (MAPbI_3_), incorporating a 5-ammonium valeric acid (AVAI) additive, forming (5-AVA)_*x*_MA_1−*x*_PbI_3_. AVAI has been found to be crucial for improving performance and stability in CPSCs.^20^

Different recombination mechanisms may dominate in different PSC architectures, depending on the perovskite layer quality, transport layers, interfacial properties, *etc.* In CPSCs, the *m*-TiO_2_ region provides an extended interface which results in high non-radiative recombination throughout the bulk mixed perovskite-oxide medium, which appears to be the main obstacle to improve the open circuit voltage (*V*_OC_).^21^ Grain boundaries due to the limited pore sizes in *m*-ZrO_2_,^22^ the interface between *m*-TiO_2_ and *m*-ZrO_2_,^23^ as well as the non-selective interface with the carbon-based back contact,^24^ have also been suggested to be significant recombination sources. In this work we show how ionic screening can prevent the collection of charges photogenerated in the *m*-ZrO_2_ region. Combining experiments and simulations we demonstrate that the electrostatic effect of mobile ions causes current collection losses and the characteristic bump in *J*–*V* curves of CPSCs, due to a screening effect which can be visualised using external quantum efficiency (EQE) measurements.

## 2 Results and discussion

### 2.1 Current losses in CPSCs

While the bump occurs in planar architectures also, even in a p–i–n configuration (Fig. S3), we chose CPSCs as study object because it is strongly pronounced and common in these devices, where it is relevant for considerable losses and still under discussion.^11,12,25–28^*J*–*V* curves of a CPSC under AM 1.5 G solar simulator illumination, with varying scan rates, are shown in Fig. 1b. These are measured from a *V*_OC_ precondition, starting with the backward scan. The backward (BW) and forward (FW) scans are shown separately in Fig. 1c, zoomed in on the bump. The hysteresis area, calculated as the total difference between the BW and FW scans, is plotted in Fig. 1d for *J*–*V* curves measured with different scan rates, under white LED illumination. The *J*–*V* curves show increased *J*_SC_ loss with decreasing scan rate (Fig. 1f), a hysteresis area which peaks at an intermediate scan rate of ≈200 mV s^−1^, and a transition towards a lower *J*_SC_ seen most obviously as a bump in the BW scan, which occurs at higher/earlier applied voltages for lower scan rates. The relative bump height, calculated as the difference between the maximum current along the *J*–*V* curve and the *J*_SC_, is also plotted in Fig. 1d for the different scan rates. The peak hysteresis area occurs at higher scan rates than the peak bump height in the BW scan, indicating that the relaxation process leading to the reduction in current happens predominantly during the FW scan for faster scan rates. This results in the highest hysteresis level being reached prior to significant *J*_SC_ losses in the BW scan. Fig. 1c clearly illustrates this effect; for the *J*–*V* at 100 mV s^−1^, the reversal of the scan direction causes an abrupt change with a fast transition to low currents. This transition becomes less abrupt and slower when it starts during the BW scan for slower scan rates. As shown in Fig. 1e (and Fig. S2 showing all *J*–*V*s), the bump in *J*–*V* curves exhibits a dependence on illumination wavelength. The reduction in bump height with decreasing illumination wavelength, resulting in lower *J*_SC_ loss (relative to the fastest scan) is shown in Fig. 1f. Since light of shorter wavelengths is predominantly absorbed closer to the surface, and longer wavelengths deeper in the absorber, this indicates that the transient process leading to the bump and *J*_SC_ loss makes it increasingly difficult to collect charges the deeper in the absorber they are generated.

**Fig. 2:**
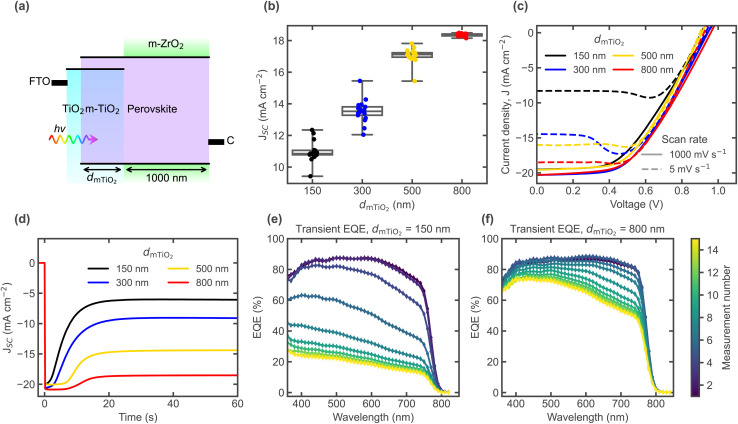
(a) Representative energy level schematic of CPSCs. Light is illuminated from the FTO side, photogenerated electrons are collected by the mesoporous ETL, whereas holes are transported through the perovskite all the way to the carbon-based electrode. The *m*-TiO_2_ thickness is varied while keeping the *m*-ZrO_2_ constant. (b) Steady state *J*_SC_ values from *J*–*V* curves measured at slow scan rate (1 mV s^−1^) right after fabrication, comparing different thicknesses. (c) *J*–*V* curves comparing scan rates. (d) Transient *J*_SC_ upon a change from OC (10 s) to SC conditions. Transient EQE spectra for cells with (e) 150 nm and (f) 800 nm *m*-TiO_2_, recorded at low temperature (≈−30 °C) after cooling down under 1 V and switching to SC. Each individual measurement takes around 4 min.

To better understand the cause of current losses, triple mesoscopic CPSCs with different oxide-perovskite layer thicknesses were measured. The *J*_SC_ obtained for various *m*-TiO_2_ layer thicknesses is shown in Fig. 2. In this case the thickness of the *m*-ZrO_2_ was kept constant (1000 nm), as depicted in Fig. 2a. The *J*_SC_ values in Fig. 2b originate from *J*–*V* curves measured shortly after fabrication, all at the same slow scan rate (1 mV s^−1^) with a solar simulator. In agreement with previous reports, thinner *m*-TiO_2_ layers result in lower *J*_SC_. Liu *et al.*^21^ argued that the photocurrent generated at the *m*-ZrO_2_ layer could not be collected, which is why a thicker *m*-TiO_2_ layer is needed. Variations of the scan-rate reveal a fuller picture: with a fast measurement (1000 mV s^−1^) starting from open circuit (OC), the difference between the different thicknesses is significantly smaller (Fig. 2c). This is better illustrated by the transient *J*_SC_ upon switching from OC to short circuit (SC) as seen in Fig. 2d; the initial level is similar but the stabilised level differs greatly. The transient process behind this current loss has been discussed in previous work and attributed to the electric field screening effect of mobile ions.^29^ The dependence on the scan rate shown in Fig. 2c indicates that the effect of mobile ions also plays a role in the current scaling with layer thickness, *i.e.* in the ability to extract charges photogenerated in the *m*-ZrO_2_ region.

It should be noted that the *J*_SC_ values measured freshly after fabrication (Fig. 2b) are higher than the ones in Fig. 2c–f, since the cells were stored for several months in between these measurements – this may have resulted in some slight degradation, *e.g.* a higher density of defects, which may increase the current loss further. Additionally, the *J*–*V* curves in Fig. 2c were measured for stored encapsulated samples, whereas Fig. 2d–f shows results for stored non-encapsulated samples, which is why the slow scan-rate and steady-state transient current do not exactly match. However, this does not compromise the analysis since the discussed trends and scaling with thickness are still the same after storage, for both encapsulated and non-encapsulated samples.

As discussed before, current collection losses depend on the illumination wavelength (Fig. 1e and f), so they can be more accurately analysed with EQE measurements to check for spectral variations. At room temperature, transient ionic effects in the seconds-timescales can be hardly measured by conventional EQE, since it is generally a slow measurement. Measuring at low temperature to slow down ionic movement, enables to compare the effect of different ionic distributions.^30^ By cooling under a forward bias around the illuminated *V*_OC_ value, and then switching to SC and continuously recording EQE profiles over time, the transient spectral dependence of the ion-induced *J*_SC_ losses can be resolved. Fig. 2e and f show low temperature (≈−30 °C) EQE transients for two different *m*-TiO_2_ thicknesses (150 nm and 800 nm respectively). Integration of the transient EQE profiles to compute *J*_SC_ yields a similar transient as the *J*_SC_ measured upon switching from OC to SC (Fig. S4). In recent work, we have shown how EQE losses at long wavelengths are caused by a low electric field in the bulk away from the compact TiO_2_ surface, as a result of the screening effect of mobile ions.^30^ In brief, before mobile ions have time to redistribute to screen the electric field at SC (when preconditioning with a forward bias), a higher electric field in the bulk aids in the extraction of charges photogenerated deeper in the absorber. For a low *m*-TiO_2_ thickness as shown in Fig. 2e, the transient EQE goes down over the whole spectral range, albeit still showing a greater loss at longer wavelengths (red region) as seen by the normalised spectra in Fig. S4. In contrast, if the *m*-TiO_2_ is much thicker as shown in Fig. 2f, the EQE decreases predominantly in the red, also in agreement with the current losses in Fig. 1f for a cell with 500 nm *m*-TiO_2_ thickness. Thus, for efficient charge collection, the thick *m*-TiO_2_ layer where bulk electron injection can take place seems primarily a necessity caused by the screening effect of mobile ions, which can be overcome by an adequate distribution of the mobile ionic charge.

**Fig. 3:**
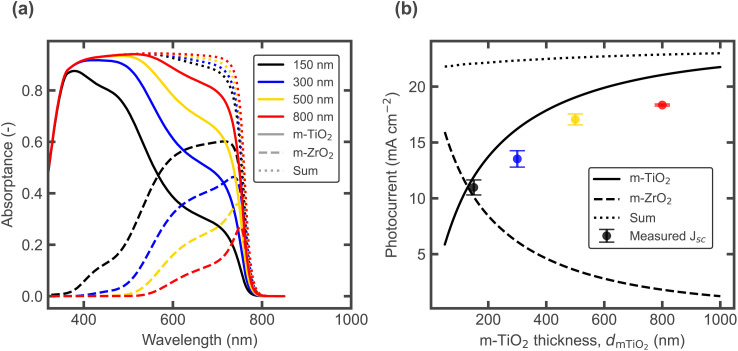
(a) Simulated layer absorptance for varying *m*-TiO_2_ thickness, with a constant 1000 nm *m*-ZrO_2_ layer, using an effective medium approximation with 65% MAPbI_3_ and 35% oxide. (b) Simulated 1 sun photocurrent (sum) and contribution from each layer, plotted together with the experimental steady state *J*_SC_ values from Fig. 2b.

Fig. 3 shows optical simulations (carried out using Setfos with the parameters provided in the SI) using an effective medium approximation to calculate the complex refractive index of the mixed perovskite-oxide layers, assuming volume fractions of 65% MAPbI_3_ and 35% oxide (TiO_2_ or ZrO_2_). The absorptance of each layer is shown in Fig. 3a, for devices with the different *m*-TiO_2_ layer thicknesses measured experimentally. When comparing the computed total photocurrent and the contribution from each oxide layer, the measured *J*_SC_ mostly follows the contribution from the *m*-TiO_2_ region (Fig. 3b), confirming that the lower stabilised *J*_SC_ values mainly originate from the inability to collect the *m*-ZrO_2_ photocurrent after mobile ions respond. Fig. S5a and b shows the same optical simulations and comparison with experimental *J*_SC_, now for a fixed *m*-TiO_2_ thickness of 500 nm, with varying the *m*-ZrO_2_ thickness. Increasing the *m*-ZrO_2_ thickness increases the *J*_SC_ and brings it closer to the sum of the photocurrents (up to a maximum at 2000 nm after which the series resistance likely affects *J*_SC_), indicating that the non-selective carbon contact also plays a crucial role. The experimental EQEs as a function of *m*-ZrO_2_ thickness (Fig. S5c) further illustrate how an adequate spacing of the carbon contact can also help to minimize photocurrent loss, particularly for charges generated deeper in the cell (with light of longer wavelengths), as seen by the normalised spectra (Fig. S5d). The greater distance to the generation region seems to reduce the probability of recombination at the non-selective back contact, in agreement with the findings by Wagner *et al.*^24^

### 2.2 Electric field screening

To better understand the occurrence of the bump in *J*–*V* curves, a simplified 1D electrical device model is set up in the drift-diffusion simulation software Setfos. The model device consists of an intrinsic absorber (perovskite) between selective CTLs, where ETL and HTL parameters are fully symmetric for electrons and holes, respectively (Fig. S6). While in mesoscopic CPSCs no HTL is used, and the non-selective contact at the hole-collecting side may introduce an additional recombination source if not spaced properly, holes can still be effectively transported to the back electrode through the *m*-ZrO_2_ layer. We begin with a model as generic and simple as possible (perfectly selective), focusing primarily on the effects of ionic screening, since the *J*–*V* bump can be observed across different PSC architectures and is not exclusive to HTL-free devices^2^ (Fig. S3b–d). The scenario without a selective back-contact (HTL-free) is discussed later. The model considers the mixed ionic-electronic conductivity of perovskite by including mobile ionic charges confined to the absorber layer (for simplicity anions and cations with the same mobility and concentration). These ionic charges, assuming they induce shallow energy levels only, do not act as recombination centres themselves but only act as a dynamic doping charge which redistributes to screen the electric field. While the electric field in a solar cell is not strictly necessary for driving charge separation,^31,32^ as the actual driving force is given by the gradient of the electrochemical potential, it still serves in this case as a first useful indicator of how ionic charge redistribution impacts charge collection.

**Fig. 4:**
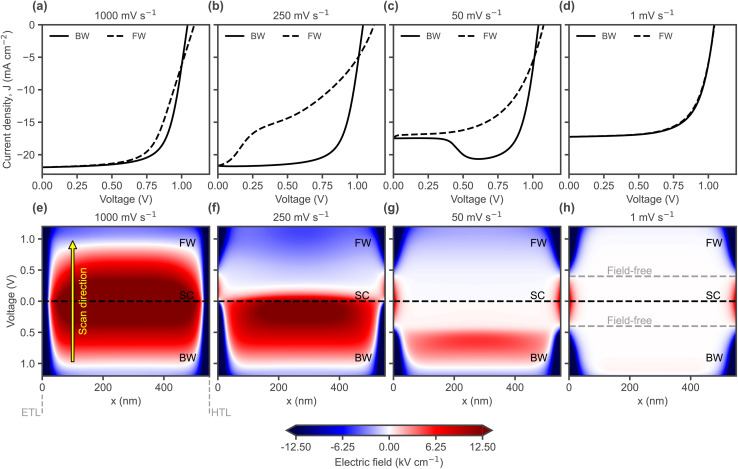
(a)–(d) Simulated *J*–*V* curves with varying scan rates, starting with the BW scan from an illuminated *V*_OC_ precondition. (e)–(h) Electric field profiles along the perovskite layer depth (*x*) during the *J*–*V* scans above, for different scan rates. Note that the voltage scan starts at 1.2 V in the BW direction, so the electric field evolves over time from bottom (BW scan) to short circuit (SC) to top (FW scan). The edges of the plots at 0 nm and 550 nm correspond to the location of the interfaces with the ETL and HTL, respectively. The energetic offset between the perovskite and CTLs is Δ*E*_CTL_ = 0.3 eV, with *V*_bi_ = 0.6 V.

Fig. 4a–d shows simulated scan rate dependent *J*–*V* curves starting with the BW scan from a steady state precondition at illuminated *V*_OC_ (full set of scan rates in Fig. S7); the bump in simulations occurs as a direct result of the electric field screening effect of mobile ions, where the ionic density and mobility influence the scan rates at which the hysteresis area and relative bump height peak (Fig. S8–S11). In simulations, the different shapes of scan rate dependent *J*–*V* curves can be understood mainly from ionic screening in the presence of a high recombination rate. Fig. 4e–h shows the evolution of the electric field along the perovskite layer during the applied voltage sweeps, for an ion mobility of 5 × 10^−10^ cm^2^ V^−1^ s^−1^ (ionic charge profiles in Fig. S12). The sign of the electric field is defined as positive when it contributes to separating electrons and holes toward their respective terminals. As shown in Fig. 4, for a fast scan rate (1000 mV s^−1^) before ions have time to redistribute, the electric field is highest at SC and the evolution is mostly symmetric with respect to BW and FW scans, with the field changing sign only at voltages higher than *V*_OC_ ≈ 1 V (Fig. 4a and e). However, with a lower scan rate (250 mV s^−1^), mobile ions are able to respond during the voltage scan and they completely screen the electric field in the bulk at around ≈0.25 V, reached shortly after having passed SC (Fig. 4b and f). This results in a *J*_SC_ which is not affected, but in a sudden drop in the current for voltages between 0 and ≈0.25 V during the FW scan. As ions redistribute (a process that takes time), the scan rate remains faster than their ability to respond, causing them to remain in a distribution that leads to a reversal of the electric field, making it negative during the FW scan – in agreement with previous explanations by Calado *et al.*^33^ and Courtier *et al.*^34^ This lagging ionic distribution (for a lower voltage than the applied one) and the resulting inverted electric field lower the collected current significantly as seen by the larger hysteresis area. Decreasing the scan rate even more (50 mV s^−1^), results in an ionic field screening response which manifests earlier, already in the BW scan. This can be seen by the highest electric field occurring at the voltage where the bump in the *J*–*V* can be seen (Fig. 4c and g). After this point, the ionic screening effect causes the electric field to drop significantly, lowering the current. At this scan rate, the difference between the ionic redistribution time and the voltage ramp time is smaller. The field remains lower after the screening response, affecting the *J*_SC_. Since the ionic response still lags behind the voltage ramp, there is still a slight switch to a negative field in the bulk during the FW scan, which causes the current to drop to lower values than seen in the quasi-steady state (slowest scan rate) where the ionic response fully follows the voltage scan. The latter situation is shown in Fig. 4d and h, where the electric field in the bulk is almost completely screened at each applied voltage, as the scan rate is now slow enough for ions to reach a steady state at each voltage level, resulting in the lower *J*_SC_ and showing no hysteresis. In essence, the bump in the BW scan (corresponding to a maximum in the bulk electric field before reaching SC) arises when the ionic response is slightly slower than the scan rate, causing it to lag but still respond within the BW scan duration.

**Fig. 5:**
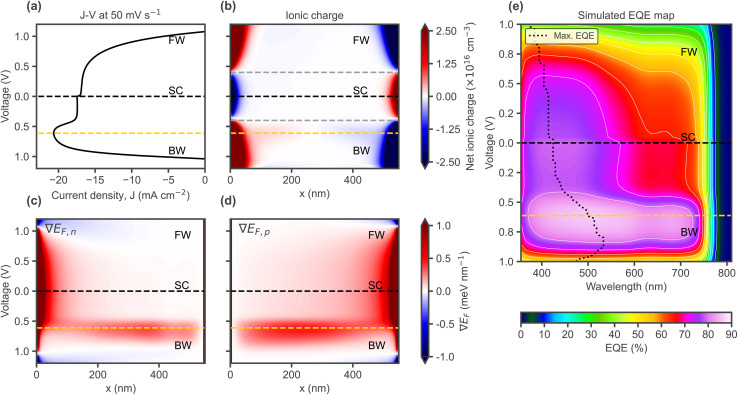
(a) Simulated *J*–*V* curve at 50 mV s^−1^ as in Fig. 4c and g, highlighting the voltage level where the current maximum occurs (dashed line in gold). (b) Transient ionic charge profiles during the voltage scan. The dashed grey lines indicate the point where ions are mostly compensated in the bulk (close to the ‘field-free’ situation in Fig. 4h). Evolution of quasi-Fermi level gradients for (c) electrons and (d) holes. Here, ∇*E*_F_ is defined as positive when it drives carriers toward their respective collecting electrodes, and negative when it drives them toward the opposite contact. (e) Evolution of the EQE during the voltage scan.

Until this point, only the electric field in the bulk has been discussed. However, it can be seen at the edges of Fig. 4e–h, representing the interfaces with the CTLs, that when the field is high in the bulk, it can be negative at the interfaces (Fig. 4e), due to the large energetic offset (Δ*E*_CTL_) and influenced by the space charge resulting from ionic accumulation (Fig. S32). Similarly, when ionic redistribution screens the field in the bulk, it remains high only close to the interfaces. The changes observed suggest a scan rate-dependent shift from bulk-to front interface-dominated spatial charge collection. Fig. 5 shows the spatial distribution of the total driving force for charge transport: the gradient of the electrochemical potential, or quasi-Fermi level (∇*E*_F,n_ and ∇*E*_F,p_ for electrons and holes, respectively), for the voltage scan where the bump is observed at 50 mV s^−1^ in Fig. 4 (the rest of the scan rates are shown in Fig. S13). The voltage level at which the current maximum in the *J*–*V* occurs is highlighted by a gold dashed line in Fig. 5a. Changes in the ionic charge (Fig. 5b) and electric field (Fig. 4g) are directly reflected in the total driving force for charge transport (Fig. 5c and d), which justifies our analysis based on electric field profiles. Notably, even when the electric field becomes negative, the total driving force still points in the desired direction but reduces accordingly, indicating that charge currents can still flow against the electrical force component, thanks to the chemical one. The bump, or decrease in current in Fig. 5a after the maximum, effectively coincides with the decrease of the driving forces in the bulk and increase close to the interfaces. The spatial changes in the driving force for charge currents can lead to differences in current collection depending on the absorption depth profile and thus the illumination wavelength, as shown by the simulated evolution of the EQE in Fig. 5e, which is experimentally accessible and can be used to visualise the shifts in collection efficiency along the depth of the absorber (caused by ionic screening), as discussed in the next section. The EQE simulations were carried out by perturbing the incident AM 1.5G illumination spectrum at different wavelengths (using a small-signal approach) for the different combinations of applied voltage and ionic space charge distribution corresponding to the different data points along the BW and FW sections of the transient *J*–*V* curve.

### 2.3 EQE mapping

**Fig. 6:**
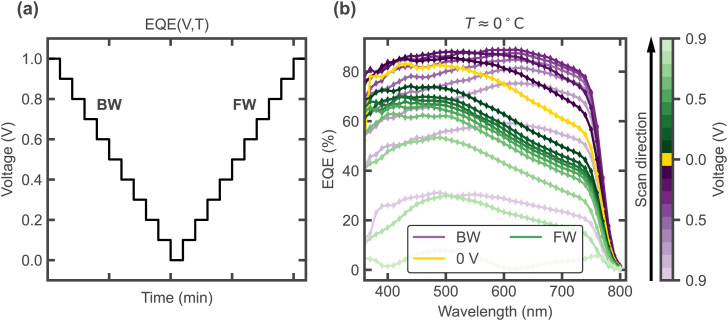
(a) Measurement procedure, recording EQE spectra at each bias voltage level in a staircase order, where each EQE measurement takes ≈2–3 min. (b) Experimental EQE spectra measured under different bias voltages in order, starting with the BW scan from a forward bias precondition (cooling to 0 °C under 1 V).

**Fig. 7:**
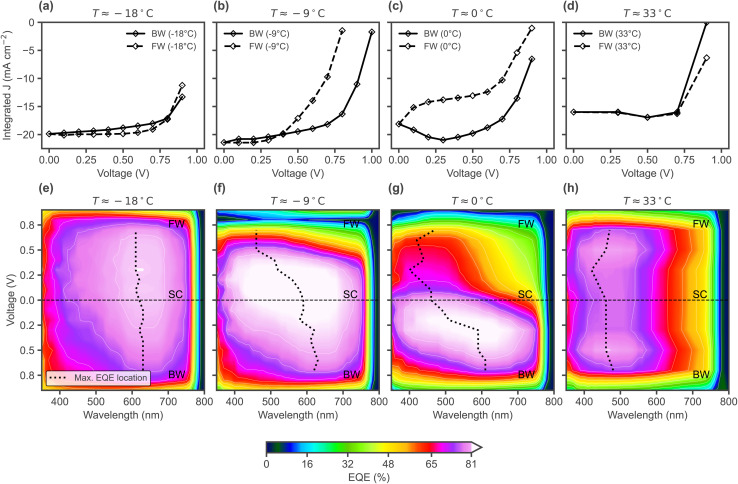
(a)–(d) Experimental *J*–*V* curves reconstructed from the EQE(V) spectra (using 1 sun AM 1.5G spectrum for the integration), measured under different temperatures, starting with the BW scan from a precondition at 1 V. (e)–(h) Contour maps of EQE-V profiles for different device temperatures, showing the wavelength location of the maximum EQE at each bias voltage.

As demonstrated in Fig. 5e, the effects of the changes seen in the simulated electric field profiles in Fig. 4e–h may be visualised experimentally *via* voltage dependent EQE measurements. By preconditioning at a bias level around *V*_OC_ (as done to measure *J*–*V* curves) and then measuring EQE(V) in a staircase order, spectral changes during the voltage scan can be visualised. While the experimental setup does not allow for a direct control of the staircase scan rate (Fig. 6a), varying the temperature allows to compare different rates of ion migration, which is effectively comparable to varying the scan rate. Fig. 6b shows EQE(V) for a temperature of around 0 °C. Fig. 7a–d shows the reconstructed *J*–*V* curves from the EQE(V) spectra (taken at different temperatures), which show the same four types of curves as the simulated ones in Fig. 4a–d, corresponding to different scan rates. Furthermore, Fig. 7e–h shows the spectra plotted as contour maps to visualise EQE-V for the different scan rates/temperatures. Consistent with the trends in the simulated electric field spatial maps shown in Fig. 4e–h, lowering the scan rate (*i.e.* increasing temperature in experiment) causes the EQE maximum (dotted line) to shift from longer to shorter wavelengths during the scan due to ionic screening. This shift does not occur for −18 °C (corresponding to a fast scan rate, Fig. 7e). For −9 °C (intermediate scan rate, Fig. 7f), the EQE drops and its peak wavelength shifts during the FW scan, resulting in high hysteresis in agreement with the bulk electric field inversion from Fig. 4 discussed before. At 0 °C (slow scan rate, Fig. 7g), the overall maximum EQE and subsequent decrease and spectral change occurs already during the BW scan, as also seen in the simulated EQE map in Fig. 5e, leading to the characteristic bump in the BW current. Finally, for the highest temperature (mimicking the slowest scan rate), the EQE is overall lower and the maximum consistently occurs at shorter wavelengths (Fig. 7h). While we have shown that the effects of voltage scan rate-dependent ionic transients can be spectrally resolved using EQE mapping, transient ion drift during single EQE measurements may occur. This effect, inherent to slow conventional EQE measurements, could be mitigated by using flash-EQE. Nevertheless, any potential slight distortion of the individual spectral profiles is not expected to affect the overall measured trends, which remain in full agreement with the simulated profiles.

### 2.4 CTL properties and recombination mechanisms

The explanation provided so far does not clarify why some PSCs show the bump in the BW *J*–*V* scan whereas others do not, as seen in the results by Thiesbrummel *et al.*^7^ for instance, where planar p–i–n cells show no bump despite the *J*_SC_ and hysteresis also exhibiting a scan rate dependence due to ionic screening. The scan rate at which hysteresis peaks, can differ by orders of magnitude depending on the CTLs used, especially depending on whether an organic layer or inorganic oxide layer is used as CTL; Levine *et al.*^5^ showed how different CTLs, as used in p–i–n or n–i–p PSCs with the same absorber, can determine the rate of ion migration of the system (as seen by the peak hysteresis scan rate). Courtier *et al.*^34^ explored these differences in simulations, identifying the bump as a feature of significant bulk recombination and also highlighting how CTL parameters critically affect the peak hysteresis scan rate. Specifically, a larger potential drop across the CTLs occurs with lower CTL doping (or permittivity), which shifts the hysteresis peak to faster scan rates as the density of ionic charge displaced for internal field screening is reduced.^34,35^ More recently, Wang *et al.*^13^ assigned the bump in simulated *J*–*V* curves to dominating bulk Shockley–Read–Hall (SRH) recombination. Nevertheless, in simulations the bump may also appear with dominating interface recombination instead (Fig. S14) as also found by Idígoras *et al.*,^10^ where the higher hysteresis and the appearance of the bump were assigned to increased surface recombination at the ETL interface.

**Fig. 8:**
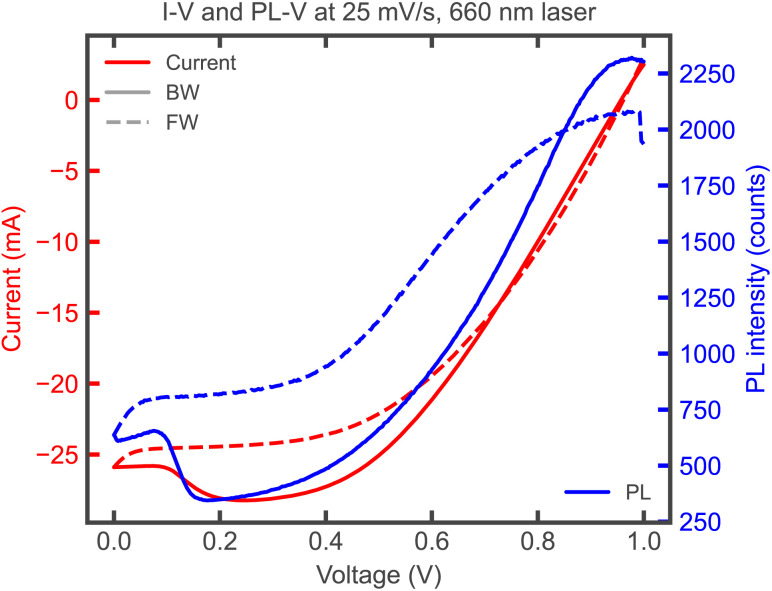
Experimental *I*–*V* and PL-V curves under illumination with a red (660 nm) laser. Axes of *I* and PL have been arbitrarily aligned.

For ionic redistribution to significantly decrease the current collected at the terminals, there must be a highly competing recombination pathway.^36^ In the ideal case, all recombination happens radiatively (band to band).^37^ Ionic screening may in fact lead to reduced current collection with increased radiative recombination, as seen in transient photoluminescence (PL) measurements upon switching from OC to SC.^29^ Therefore, it seems not possible to simply assign the bump in the *J*–*V* curve to bulk SRH, as the increased bulk recombination could also be happening radiatively as illustrated in Fig. 8, showing how the PL-V curve mirrors the *I*–*V* response (note that the PL yield is relative and can only be analysed qualitatively). The dominant recombination mechanism cannot be identified from the occurrence of the bump alone, as illustrated in Fig. S14 showing bumpy *J*–*V* curves across various types of dominating recombination. Even for the fully radiative case (Fig. S15 and S16), charge transport limitations (due to reduced electronic mobility) may also increase the scan rate-dependent *J*_SC_ loss and the bump. To clarify whether radiative or non-radiative recombination mechanisms dominate, the current loss would need to be compared quantitatively with the absolute PL yield, as both bulk radiative and SRH recombination may increase with screening (albeit differently depending on the charge carrier densities present). Furthermore, in HTL-free CPSCs the non-selective back contact can act as an additional recombination source if not spaced properly. In the drift-diffusion model, removing the HTL may introduce a major pathway for recombination. As a result the bump can be observed for the HTL-free situation without considering any bulk SRH recombination (Fig. S14c). This occurs because the non-selective contact becomes the main channel for current losses, with ionic screening resulting in enhanced losses, now *via* surface recombination (similar to Fig. S14b).

**Fig. 9:**
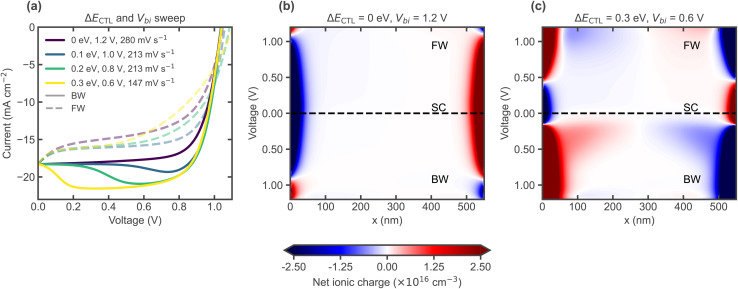
(a) Simulated transient *J*–*V* curves for various energetic level configurations, showing the same *J*_SC_ loss due to ionic screening, which occur at slightly different scan rates as the energetic alignment also influences the *J*–*V* hysteresis timescales. Δ*E*_CTL_ and *V*_bi_ are varied in parallel. Ionic charge profiles during the *J*–*V* scans in (a) for (b) perfectly aligned contacts with high *V*_bi_ and (c) large energetic offset with low *V*_bi_.

Energy level alignment with the CTLs has also been shown to affect *J*–*V* curve hysteresis.^38–40^ In our simulations, for the same parameter set as in Fig. 4, modifying the energy levels to obtain perfectly aligned contacts can also shift the peak hysteresis to higher scan rates, thanks to the lower ionic charge density displaced during the voltage sweep. In turn, this can also make the bump disappear altogether – while still showing the scan-rate dependent *J*_SC_ loss and hysteresis (Fig. S17–S25). This seems to hold for the HTL-free case where only the front-contact energetic alignment is varied (Fig. S26). Fig. 9a shows how the bump becomes less prominent and eventually disappears with increasing the built-in voltage (*V*_bi_) and lowering the energetic mismatch between perovskite and the CTLs (Δ*E*_CTL_). However, not all situations which lead to less ionic charge being displaced lead to a reduction in the bump; for a fixed small energy level mismatch (0.1 eV), the bump seems to become more prominent with decreasing CTL permittivity/doping (Fig. S27–S31). In such cases the electric field is higher within the perovskite – requiring more ions, shifting the peak hysteresis to lower scan rates, but now reducing the likelihood of observing the bump.

**Fig. 10:**
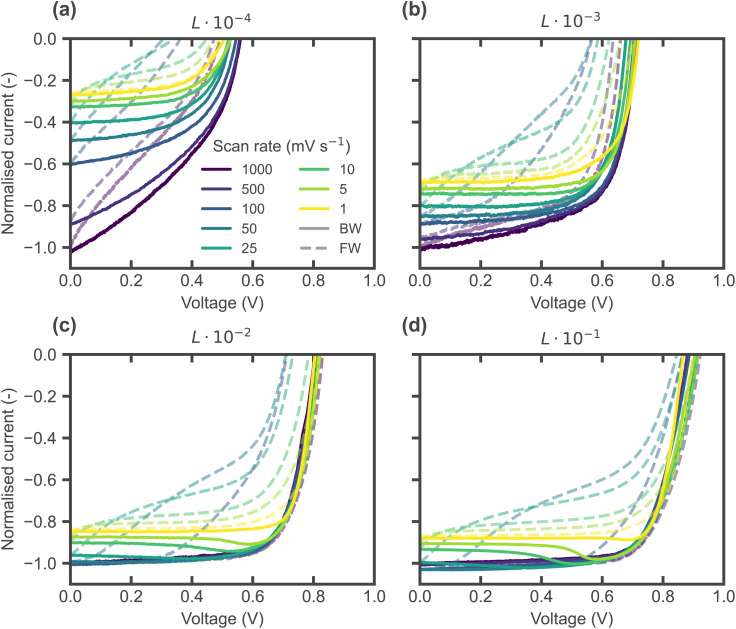
(a)–(d) Experimental light intensity-dependent *J*–*V* curves measured under white LED illumination, where *L* is the maximum light intensity of the LED, equivalent to ≈1.5 sun intensity. The *J*–*V* curves were measured starting with the backward scan from a precondition at *V*_OC_ for each scan rate. The *J*_SC_ level difference across scan rates increases with decreasing light intensity due to slow capacitive effects becoming more prominent at low intensities.

The key lies in the level of the applied voltage at which ionic accumulation layers are mostly discharged and compensated in the bulk, effectively resulting in an ‘ion-free’ situation due to the flat potential profile (no electric field), as indicated in Fig. 4h by the ‘field-free’ lines, corresponding to the levels in Fig. 5b where the ionic space charge layers change sign. This level depends greatly on the energetic alignment and CTL properties (Fig. S19, S21 and S29), as recently investigated by Hart *et al.*,^41^ and corresponds to the crossing point of steady state *J*–*V* curves for equivalent devices with and without considering mobile ions (Fig. S33 and S34). A lower field-free voltage increases the likelihood of observing the bump, as mobile ions will discharge from their interfacial accumulation at a lower voltage during the BW scan – significantly delaying the point at which the current drops due to ionic screening and resulting in a current overshoot, as can be seen by comparing the transient ionic profiles in Fig. 9b for perfect energetic alignment with those in Fig. 9c for a large mismatch. The delay in ionic redistribution can also be observed in simulations upon a change from open circuit to short circuit conditions (Fig. S22), which appears consistent with the significant delay observed experimentally in the *J*_SC_ transients in Fig. 2d, where the *J*_SC_ does not drop immediately. Furthermore, while previous work argued that the mechanism causing the bump is independent of the light intensity,^11^ the explanations above suggest that the bump should tend to disappear as *V*_OC_ approaches the field-free level, since the delay due to ionic accumulation would be prevented. Since *V*_OC_ depends logarithmically on the light intensity, it is necessary to reduce the illumination by orders of magnitude to observe this effect. As shown in Fig. 10, the bump effectively decreases with lower light intensity and indeed disappears at lower *V*_OC_ levels. In summary, the timescale, level and form of hysteresis (and whether there is a bump or not) depend on the interplay between mobile ions in the perovskite absorber, charge recombination mechanisms and contact (CTL/electrode) properties. The key ingredients to observe a bump in simulations are a high mobile ion density, high recombination rate (bulk/interface) and low ion-free (*i.e.* field-free) voltage level, for instance caused by a large energetic offset with the CTLs (Δ*E*_CTL_).

### 2.5 Implications for CPSCs

Ionic screening causes significant current collection losses in CPSCs and the transient process can be observed, at slow scan rates, as a current maximum before SC (bump) in the BW scan of *J*–*V* curves. Upon ionic screening, charges generated deeper in the absorber become more likely to recombine. The shorter path to electron injection in the ETL in the *m*-TiO_2_ region, results in most of the photocurrent loss originating from the contribution of the *m*-ZrO_2_ region – where ionic screening has the greatest impact. As discussed by Wagner *et al.*,^24^ the optimum *m*-TiO_2_ thickness should cover the charge generation depth, and at the same time the *m*-ZrO_2_ should be thick enough to minimize the detrimental effect of the non-selective back contact. However, since the radiative recombination yield (thus *V*_OC_) in CPSCs is still far from its achievable maximum, further optimisation of the cell design is needed to achieve the highest PCE possible. In this regard, increasing the *m*-TiO_2_ thickness, thus the probability of non-radiative recombination at the *m*-TiO_2_/perovskite interface, is only beneficial up to a certain thickness.^21,24^ Until now, loss analyses and optimisation efforts have overlooked ionic effects in CPSCs^22^ – which play a critical role as demonstrated in this work. Differences in collection probability for the two mixed oxide-perovskite layers can be effectively analysed *via* EQE measurements with varying voltages and preconditions to see the effect of the ionic distribution. Furthermore, the occurrence of the bump, as seen in simulations, is linked to a delay caused by the polarity inversion of the ionic space charge as the applied voltage is decreased, in line with previous explanations by Valastro *et al.*^28^ using a 1D model specific to CPSCs. This could be influenced by a large energetic offset with the TiO_2_, resulting in a high electron density in the TiO_2_ and high cation accumulation at the interface under a forward bias. In some cases, despite screening effects, the presence of mobile ions may enhance overall steady-state performance (Fig. S33 and S34), provided they are shallow defects only. This aligns with Hart *et al.*,^41^ where it was shown that mobile ions can help to realise efficient solar cells with large energetic offsets at the contacts. It is therefore crucial to consider the role of mobile ions when designing and optimising CPSCs, as they significantly affect performance and may determine the design needs of the device.

## 3 Conclusions

The bump in the backward scan of the *J*–*V* curve seen for carbon-based triple mesoscopic PSCs has been explained purely in terms of ionic effects: mobile ionic charge screens the electric field, lowering the total driving force for the separation of photogenerated electrons and holes to their respective terminals, thus increasing their chance to recombine instead of being collected. Ionic relaxation is a transient process, as such its effect is different depending on the scan rate of the applied voltage. Considering scan rates from fastest to slowest from a high forward bias precondition, initially ions have no time to respond. Once they can respond, their effect is first seen in the forward scan (later in time), where the inversion of the bulk electric field leads to the highest hysteresis. This relaxation process is also responsible for the bump in the backward scan; when lowering the scan rate further, ions can respond earlier already during the backward scan, reducing the current before reaching short circuit. However, depending on the degree of ionic accumulation around *V*_OC_, some PSCs may show the ion-induced current loss and hysteresis due to bulk field inversion in the forward scan, albeit without showing the bump in the backward scan. This is highly influenced by the field-free voltage level at which ions are mostly compensated in the bulk, which in turn depends on the CTL properties and energetic alignment of the device. Finally, a steady state *J*–*V* curve is obtained once the ionic response is faster than the scan rate. The changes in the driving force for charge currents, as a result of ionic redistribution, result in variations of the spatial collection probability, as inferred from EQE measurements. Temperature and voltage dependent EQE measurements have been used to visualise the internal energetic changes governing the hysteretic properties of rate-dependent *J*–*V* curves, confirming the trends seen in drift-diffusion simulations. Under a positive prebias around *V*_OC_, ions accumulate (positive space charge at the electron-collecting contact, negative at the hole-collecting contact), resulting in field inversion at the interfaces and collection being highest in the bulk. As mobile ions redistribute and screen the bulk electric field (the polarity of the ionic space charge layers inverts), collection shifts to being highest at the interfaces. The delayed screening of the field in the bulk occurring at low voltages in the BW scan produces the characteristic bump, as visualised in EQE spectra. This new characterisation approach and the results obtained are useful and relevant for all PSC architectures.

## 4 Methods

### 4.1 Materials

The fabrication details regarding the fabrication of devices with the structure FTO/TiO_2_/*m*-TiO_2_-5-AVA-MAPbI_3_/m-ZrO_2_-5-AVA-MAPbI_3_/C, with various combinations of layer thicknesses, can be found in the SI.

### 4.2 Measurements

*J*–*V* curves under solar simulator illumination were taken with a potentiostat (BioLogic SP-300) and a class ABB AM 1.5 G solar simulator (LCS-100, 94011A, ORIEL, USA). *J*–*V* curves with varying LED illumination wavelengths and light intensities were recorded using Paios equipped with an automated measurement table (Fluxim AG). EQE measurements were taken on an in-house built setup using a halogen lamp, a monochromator and a silicon reference diode. A lock-in amplifier was used to detect the signal at a light chopper frequency of 330 Hz, under different bias voltage levels. Temperature dependent measurements were performed with a Linkam cryostat stage. Transient EQE spectra as in Fig. 2e and f were recorded sequentially, with each complete spectrum requiring approximately 4 minutes of measurement time. The sample temperature was set to slow the evolution sufficiently, ensuring minimal spectral variation within a single measurement, while remaining high enough to observe clear temporal evolution across sequential measurements within a few hours. The solar simulator and EQE setup were calibrated with a reference silicon solar cell (RS-ID-2, FHG-ISE). PL measurements were obtained using a spectrometer (Andor Kymera 193i) with iDus 420 1016 Si CCD, with Coherent OBIS 660 nm laser excitation.

For the *J*–*V* curve reconstruction in Fig. 7 from EQE, defined as the ratio of the number of collected charge carriers to the number of incident photons at a certain wavelength and bias voltage, the photocurrent density *J*(*V*) is obtained by integrating the EQE spectra over the incident solar spectrum:
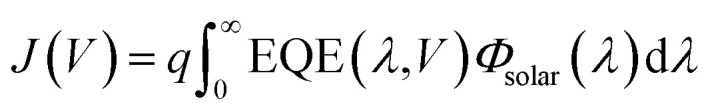
where *Φ*_solar_(*λ*) represents the solar photon flux.

### 4.3 Simulations

Device simulations were carried out using the drift-diffusion simulator software Setfos (Fluxim AG). The parameters used and details of the sweeps performed are given in the SI.

## Author contributions

M. A. T. C. conceived the study, performed the experimental measurements, drift-diffusion simulations and wrote the manuscript, under the supervision of F. N. and W. T. S. N. fabricated the samples. All the authors discussed the results and revised the manuscript.

## Conflicts of interest

There are no conflicts to declare.

## Supplementary Material

EL-001-D5EL00133A-s001

## Data Availability

The data supporting this article have been included as part of the SI. The SI provides device fabrication details, additional experimental data, the table of parameters used in simulations, and additional simulation results including parameter sweeps. See DOI: https://doi.org/10.1039/d5el00133a.
