# Supplementary material for: Visualising ionic screening in perovskite solar cells: a bumpy ride along the J–V curve
Source: EES Solar. 2025 Aug 27;1(5):762–74. doi: 10.1039/d5el00133a (PMC12394794; doi:10.1039/d5el00133a)
Supplement: EL-001-D5EL00133A-s001 [file EL-001-D5EL00133A-s001.pdf]

Supplementary Information (SI) for EES Solar.
This journal is © The Royal Society of Chemistry 2025

Supporting Information (SI) for **Visualising Ionic Screening in Perovskite Solar Cells: A Bumpy Ride Along the J-V Curve**

Miguel A. Torre Cachafeiro*[a,b], Stéphanie Narbey[c], Beat Ruhstaller[a,d], Frank Nüesch[b,e], Wolfgang Tress*[a]

[a]Institute of Computational Physics, Zurich University of Applied Sciences (ZHAW), 8400 Winterthur, Switzerland
[b]Institut des Matériaux, École Polytechnique Fédérale de Lausanne (EPFL), 1015 Lausanne, Switzerland
[c]Solaronix SA, 1170 Aubonne, Switzerland
[d]Fluxim AG, 8400 Winterthur, Switzerland
[e]Laboratory for Functional Polymers, Swiss Federal Laboratories for Materials Science and Technology (Empa), 8600 Dübendorf, Switzerland.

***Corresponding authors.** Email: miguel.torre@zhaw.ch, wolfgang.tress@zhaw.ch

May 16, 2025

1 Device fabrication details

The following details are adapted from ref. [1], with the exception that the perovskite was inkjet printed in this study, as described below.

Fluorine-doped tin oxide glass substrates (TCO22-7/LI), silver paste (Elcosil SG/SP), titania paste (Ti-Nanoxide T165/SP), zirconia paste (Zr-Nanoxide ZT/SP), carbon-graphite paste (Elcocarb B/SP) and methylammonium lead iodide perovskite solution with 5-ammonium valeric acid additive were provided by Solaronix SA. Acetone and isopropanol were purchased from Carl-Roth, ethanol was purchased from Alcosuisse. Titanium diisopropoxide bis (acetylacetonate) (75% in isopropanol) and Hellmanex were purchased from Sigma-Aldrich.

Devices were fabricated on $10 \times 10\,\mathrm{cm}^2$ FTO-coated glass plates. First, the cathode and anode areas were defined using an automated fiber laser. The substrate was then cleaned through sequential steps in a 1% aqueous solution of Hellmanex, followed by acetone and isopropanol, each for 20 minutes in an ultrasonic bath, and finally dried in air. The thin compact titania layer (c-TiO_2) was deposited by spray-pyrolysis on a hot plate heated to 550°C, with a glass mask protecting the contact areas. A solution of titanium diisopropoxide bis(acetylacetonate) diluted in absolute ethanol (1:160) was sprayed using oxygen as a carrier gas (total volume: 20 mL). The substrate was then kept warm for 30 minutes before being allowed to cool.

The screen-printing pattern enables the production of 18 cell electrodes per plate, ensuring consistency across all prints. The same screen characteristics were maintained throughout the process. The following mesh stencils were used: 100–40 for silver, 165–30 for mesoporous TiO_2 (m-TiO_2), 90–48 for mesoporous ZrO_2 (m-ZrO_2), and 43–80 for carbon pastes. To adjust the thickness of the m-TiO_2 and m-ZrO_2 layers, two approaches were employed: the pastes were diluted to obtain thinner layers, and/or multiple print passes were performed to achieve thicker layers

After printing, each wet film was left to dwell for 10 minutes before being dried at 120°C for another 10 minutes. This was followed by a firing step at 500°C (or 400°C for carbon) for 30 minutes, with a 30-minute temperature ramp. The methylammonium lead iodide perovskite precursor solution, incorporating 5-ammonium valeric acid (5-AVAI) as an additive, was selectively deposited onto the designated areas via inkjet printing, using 10 pL droplets at a spatial resolution of 1200×1200 dpi. The freshly printed samples were then annealed at 50°C for 10 minutes on a hot plate, allowing the perovskite crystals to form within the porous electrode structure. The solar cells were then individualised by cutting the glass substrate accordingly. The resulting devices underwent heat and humidity treatment at 40°C and 75% relative humidity for 135 hours, following the method previously reported by ref. [2]. Finally, encapsulation was performed by laminating a Surlyn frame around each cell, sealing it with an inert rear glass to ensure protection during characterization.

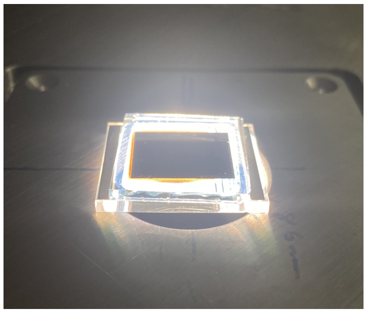

Figure S 1: Picture of encapsulated CPSC under solar simulator illumination. The active area of the device is approximately $1.45\,\mathrm{cm}^2$.

2 Experimental results

Scan rate-dependent J-V curves with varying LED wavelength illumination:

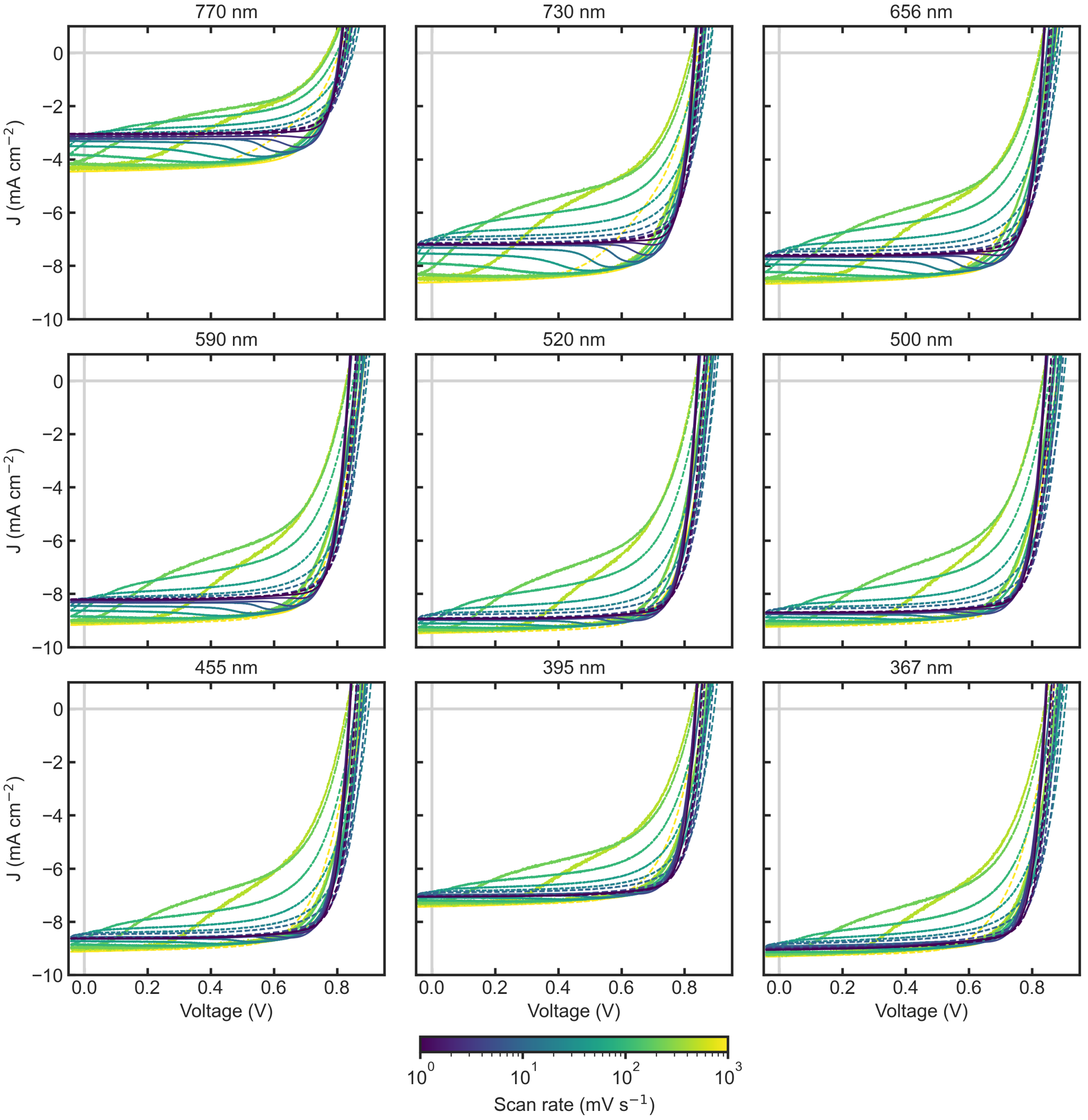

Figure S 2: J-V curves for different LEDs, for a triple mesoscopic CPSC (500 nm m-TiO_2, 1000 nm m-TiO_2). For the bump height and current loss calculations the current was normalised with respect to the J_{SC} of the fastest scan rate, for each LED, to facilitate direct comparison across the different LEDs. The photon flux of each LED was adjusted to result in a J_{SC} within a similar range for all LEDs. Small variations arise from the EQE response of the cell or from some LEDs not being capable to reach the specified photon flux, but the bump height or current loss are not expected to scale within these variations.

Bumpy J-V curve features:

Fig. 3 presents some examples of 'bumpy' J-V curves across different device architectures. Fig. 3a shows the characteristic features analysed in the main text, using example J-V curves from a C-PSC. Fig. 3b shows scan rate-dependent J-V curves for a higher efficiency fully planar p-i-n sample, comprised of FTO / Meo-4PACZ / Perovskite / Piperazine Dihydriodide / PCBM / BCP / Au. The perovskite layer was a triple cation mixed halide with composition $(FA_{0.95}MA_{0.05})_{0.95}\,Cs_{0.05}\,Pb\,(I_{0.95}Br_{0.05})_3$. In this case a bumpy feature can also be observed, as seen by the zoom in Fig. 3b. After ≈ 300 h of aging at maximum power point under 1 sun-equivalent light intensity, the scan rate-dependent J_{SC} loss aggravates, as seen in Fig. 3c, showing J-V curves for the same p-i-n sample after aging, still showing a bumpy response. Fig. 3d shows J-V curves for a fully-planar n-i-p sample also with triple cation mixed halide perovskite, comprised of FTO / ALD-TiO_2 / SnO_2 / Perovskite / PEAI / Spiro-MeOTAD / Au. The planar n-i-p sample shown is already aged after several months of storage. The J-V curves in Fig. 3d were measured at an arbitrarily low light intensity (white LED), due to the available light source at the time of measuring. It still serves as an example where a J-V bump can be observed in a fully planar device.

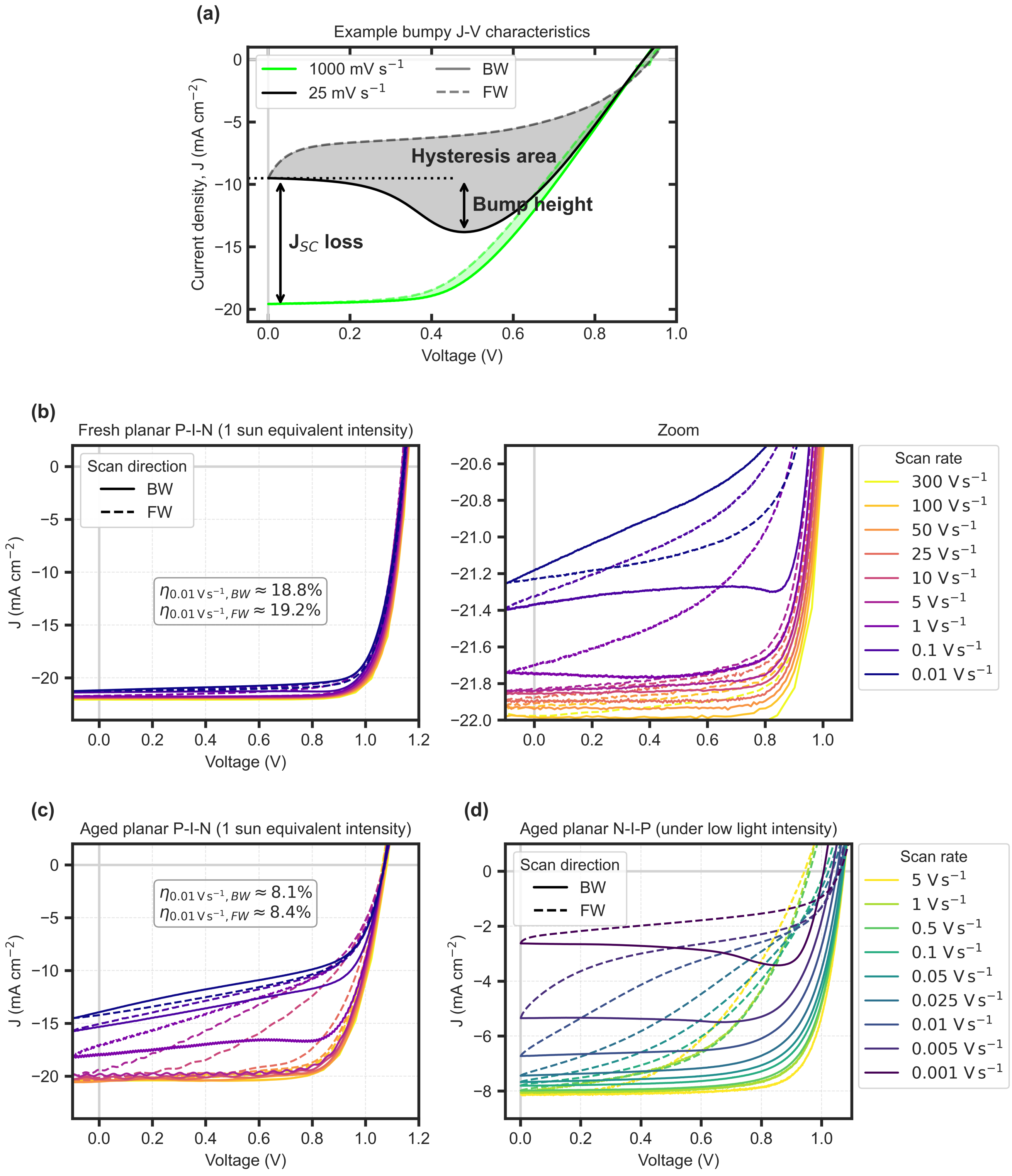

Figure S 3: (a) Schematic showing two J-V curves taken at different scan rates (starting from a V_{OC} precondition) and the definitions of the current loss (J_{SC} difference between the fast and slow scan), bump height (current difference between the maximum in the BW scan and the J_{SC}) and the hysteresis area between the BW and FW scans. (b) J-V curves for a fresh fully planar p-i-n sample and (c) same device after aging. (d) J-V curves for an aged fully planar n-i-p sample.

Transient low temperature EQE after cooling down at 1 V:

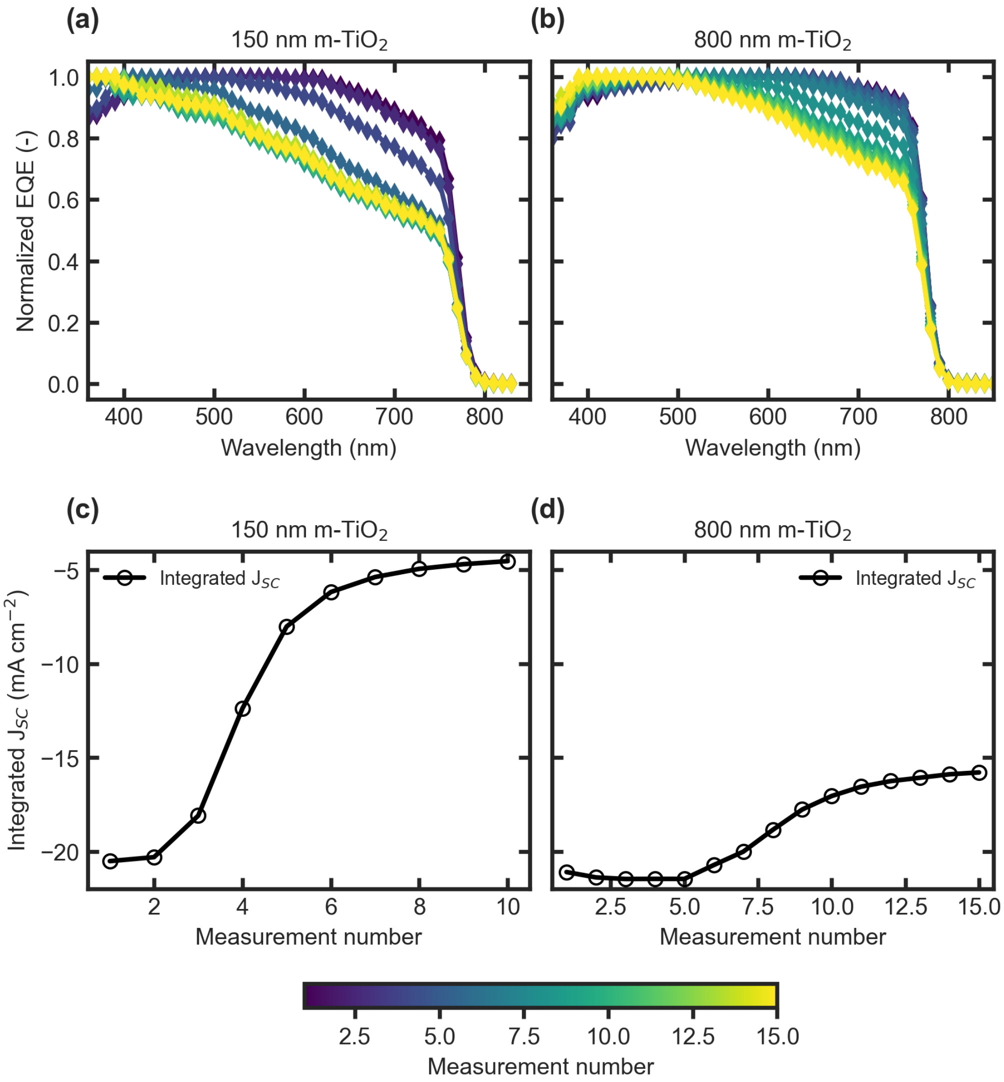

Figure S 4: (a) Normalised experimental EQE spectra of CPSCs with 150 nm m-TiO_2 and (b) 800 nm thickness, measured continuously at short circuit at low temperature after preconditioning at 1 V. The m-ZrO_2 thickness is 1000 nm in both cases. Integrated current density using the absolute EQE spectra and AM1.5G spectrum, for (c) 150 nm and (d) 800 nm m-TiO_2 thickness.

Optical simulations and comparison with experimental EQE:

The optical model uses complex refractive index (n-k) data, where the values for glass, FTO, $MAPbI_3$, ZrO_2, and carbon were sourced from the Setfos material database [3], while the n-k data for TiO_2 was obtained from [4]. A linear effective medium approximation was applied to both mixed layers, assuming 65% perovskite and 35% oxide by volume.

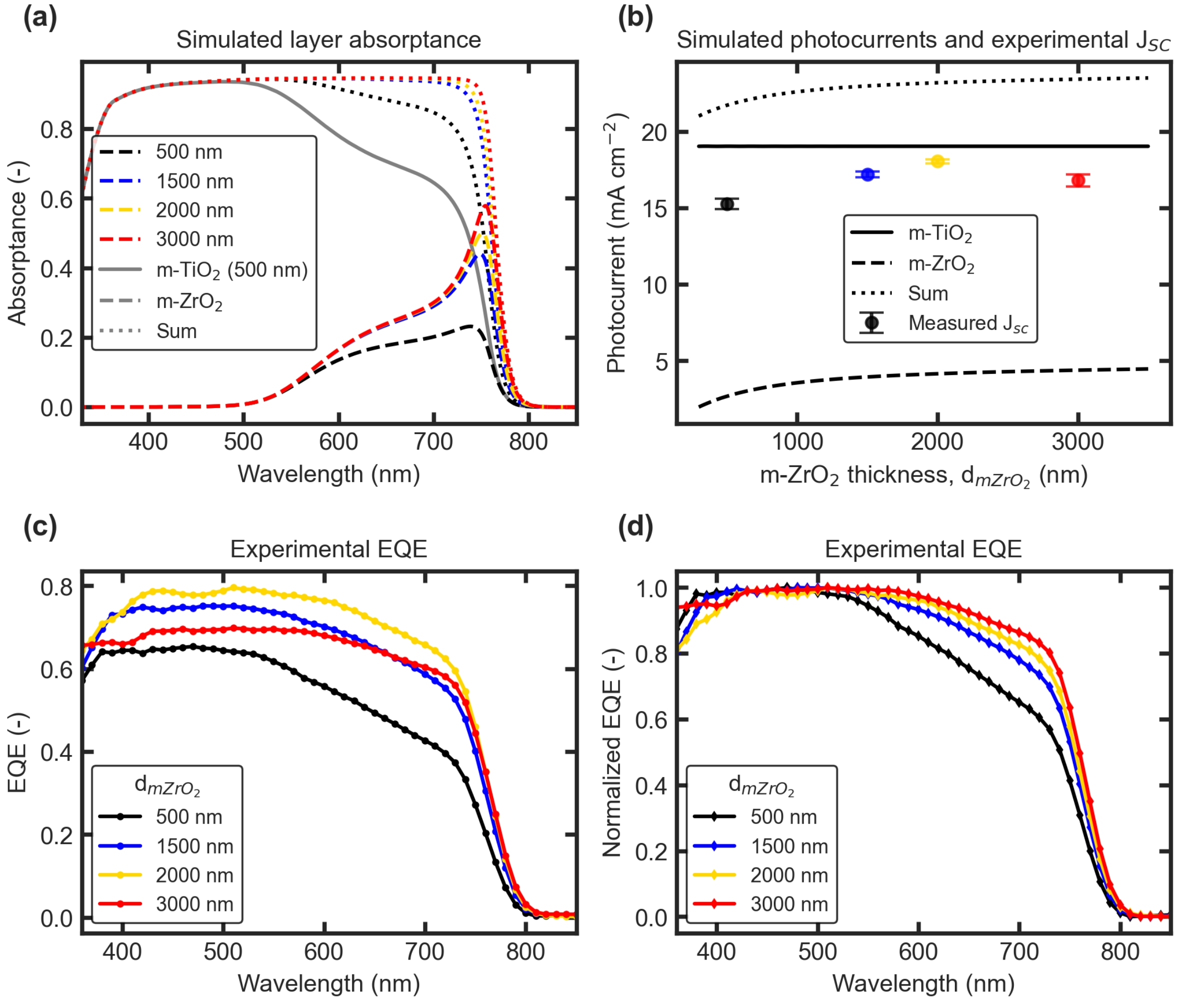

Figure S 5: (a) Simulated layer absorptance for varying m-ZrO_2 thickness, with a constant 500 nm m-TiO_2 layer. (b) Simulated 1 sun photocurrent (sum) and contribution from each layer, plotted together with the experimental steady state J_{SC} values obtained from J-V curves measured freshly after fabrication, using a slow scan rate ($1\,mV\,s^{-1}$). (c) Absolute and (d) normalised experimental EQE spectra of CPSCs with varying m-ZrO_2 thickness, measured at short circuit under room temperature.

3 Drift-diffusion simulations

3.1 Simplified PSC model parameters

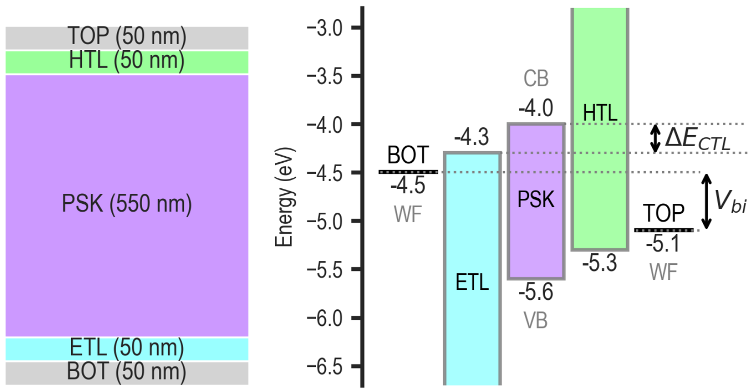

Figure S 6: (a) Simplified model consisting of a perovskite (PSK) layer between selective charge transport layers (CTLs), showing device layer stack and thicknesses. BOT and TOP correspond to the bottom and top electrodes, respectively. (b) Energy level diagram of the model. The relative energy level differences to the conduction band (CB) and valence band (VB) of the PSK layer are varied in the simulations, as well as the work function (WF) values of the TOP and BOT electrodes, which difference defines the built-in voltage (V_{bi}). The situation depicted here corresponds to $\Delta E_{CTL} = 0.3\,eV$, $V_{bi} = 0.6\,V$.

The main starting parameters used in Setfos for the fully symmetric simplified model are summarised in Table 1. Symmetry is defined relative to the left or right sides of PSK, meaning that parameters defining electron transport towards the bottom electrode are identical to parameters defining hole transport towards the top electrode. The only asymmetry in the model is introduced by the charge generation profile, which is calculated from the solar irradiation spectrum of AM1.5G coupled to an optical model which uses the complex refractive indices (n-k data) of $MAPbI_3$. The model also includes the transport of mobile ionic charges limited to the perovskite layer, with an equal concentration of opposite charges (cations and anions) with the same mobility.

Table S 1: Starting parameters used in the simplified PSC model.

Parameter	**BOT**	**ETL**	**PSK**	**HTL**	**TOP**
Thickness, d [nm]		50	550	50	
Work function, WF [eV]	4.5				5.1
Valence band energy, VB [eV]		7.5	5.6	5.3	
Conduction band energy, CB [eV]		4.3	4.0	2.1	
Density of states, DOS_{VB} [cm^{-3}]		10^{21}	10^{19}	10^{21}	
Density of states, DOS_{CB} [cm^{-3}]		10^{21}	10^{19}	10^{21}	
Electron mobility, μ_n [$cm^2V^{-1}s^{-1}$]		10^{-3}	1	10^{-3}	
Hole mobility, μ_p [$cm^2V^{-1}s^{-1}$]		10^{-3}	1	10^{-3}	
Anion mobility, μ_a [$cm^2V^{-1}s^{-1}$]			$5 \cdot 10^{-10}$		
Cation mobility, μ_c [$cm^2V^{-1}s^{-1}$]			$5 \cdot 10^{-10}$		
Dielectric constant, ε		5	24	5	
Anion density, N_a [cm^{-3}]			10^{17}		
Cation density, N_c [cm^{-3}]			10^{17}		
Bimolecular rec. prefactor, β_γ [cm^3s^{-1}]			10^{-10}		
SRH lifetimes, τ_n and τ_p [ns]			35		
Interface rec. velocity, $v_{int,SRH}$ [cms^{-1}]			0		

3.2 Parameter sweeps

Transient J-V curves (with varying scan rates) for the inital parameters in Table 1:

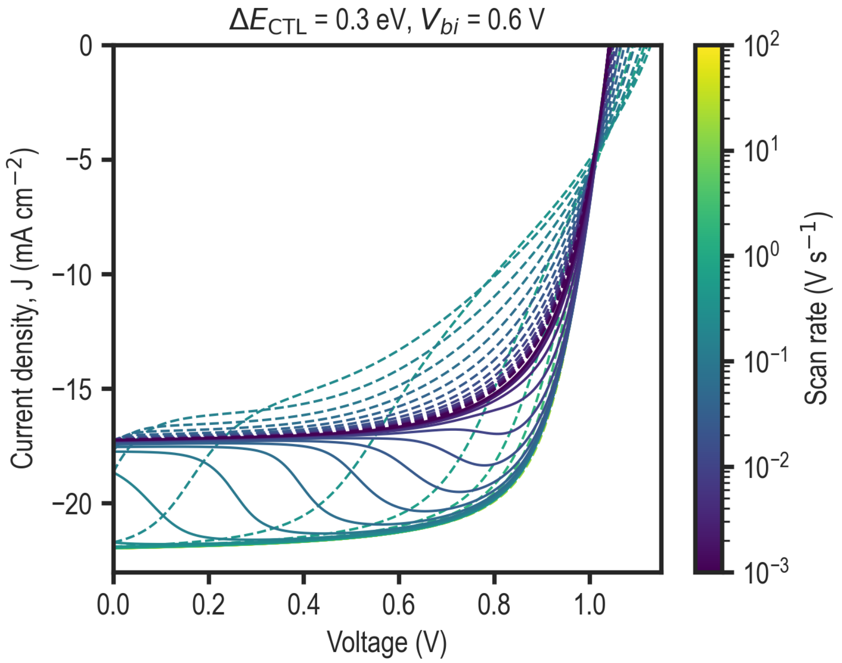

Figure S 7: Simulated J-V curves for varying scan rates. The simulated transient starts from a steady-state precondition at illuminated open circuit, with the backward scan first (solid line), followed by the forward scan (dashed line), changing direction at short circuit. In this case $\Delta E_{\mathrm{CTL}} = 0.3\,\mathrm{eV}$, $V_{\mathrm{bi}} = 0.6\,\mathrm{V}$.

3.2.1 Ionic parameters

Varying the ionic concentration $\mathbf{N_{ion}}$ ($N_a = N_c$) with fixed mobility ($\mu_{ion} = 5 \cdot 10^{-10}\,\mathbf{cm^2V^{-1}s^{-1}}$):

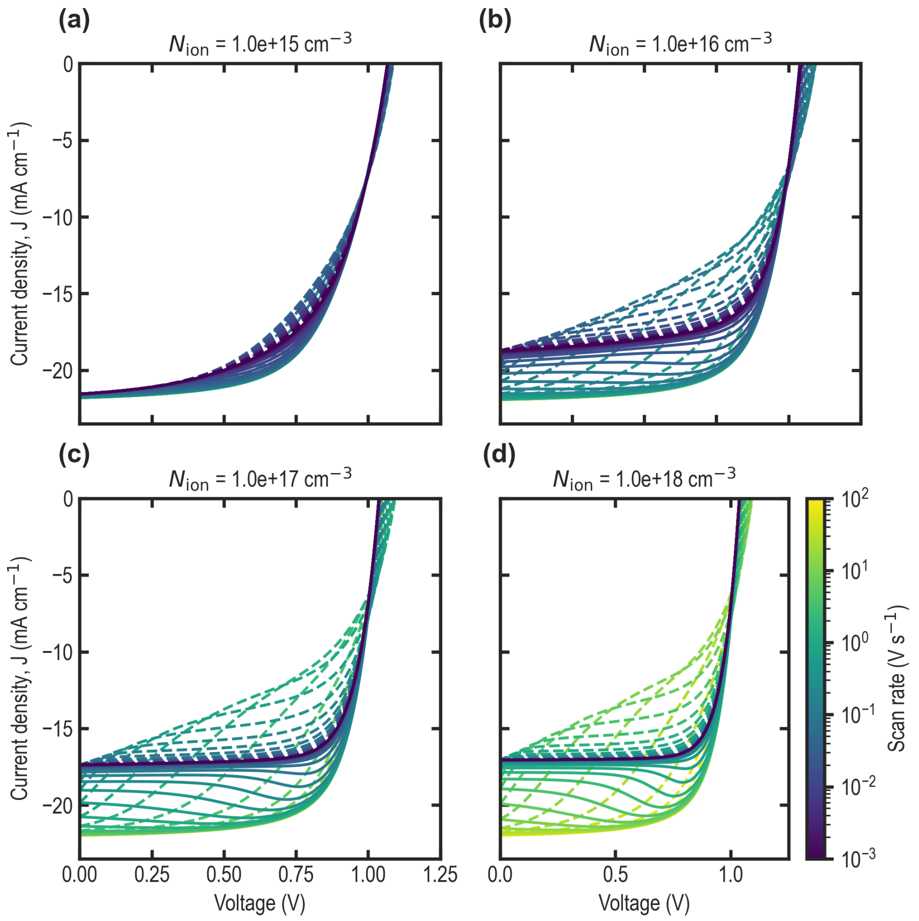

Figure S 8: (a)-(d) Simulated J-V curves for varying scan rates, with varying ion densities. The simulated transient starts from a steady-state precondition at illuminated open circuit, with the backward scan first (solid line), followed by the forward scan (dashed line), changing direction at short circuit. In this case $\Delta E_{\mathrm{CTL}} = 0.1\,\mathrm{eV}$, $\mathrm{V_{bi}} = 1.0\,\mathrm{V}$.

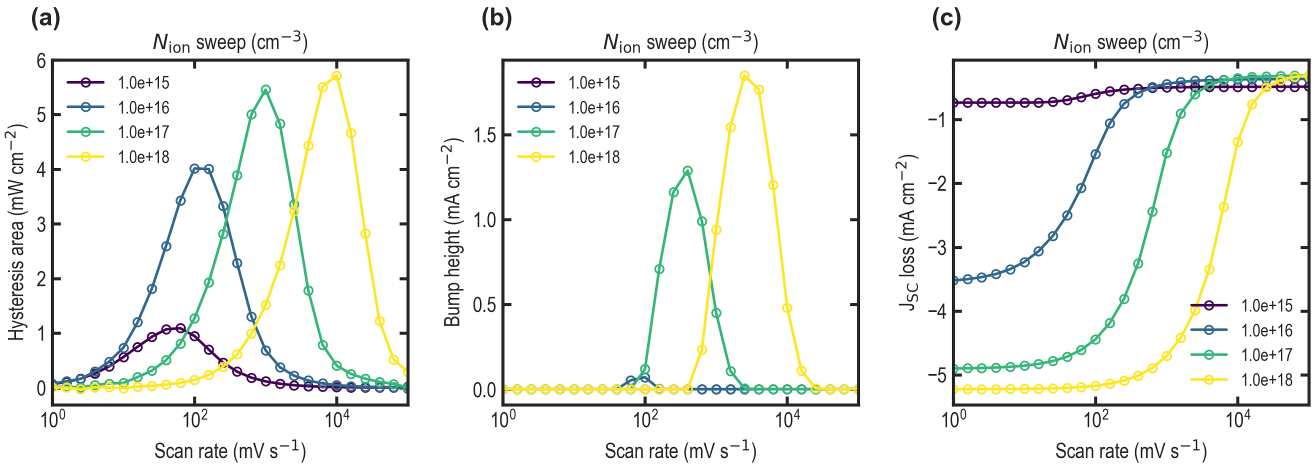

Figure S 9: (a) Hysteresis areas, (b) bump heights in the backward scan and (c) J_{SC} loss with respect to the maximum collectable photocurrent for the different scan rates, computed from the J-V curves in Fig. S8 for varying ion densities.

Varying the ionic mobility μ_{ion} ($\mu_a = \mu_c$) with fixed ion density ($N_{ion} = 10^{17}$ cm^{-3}):

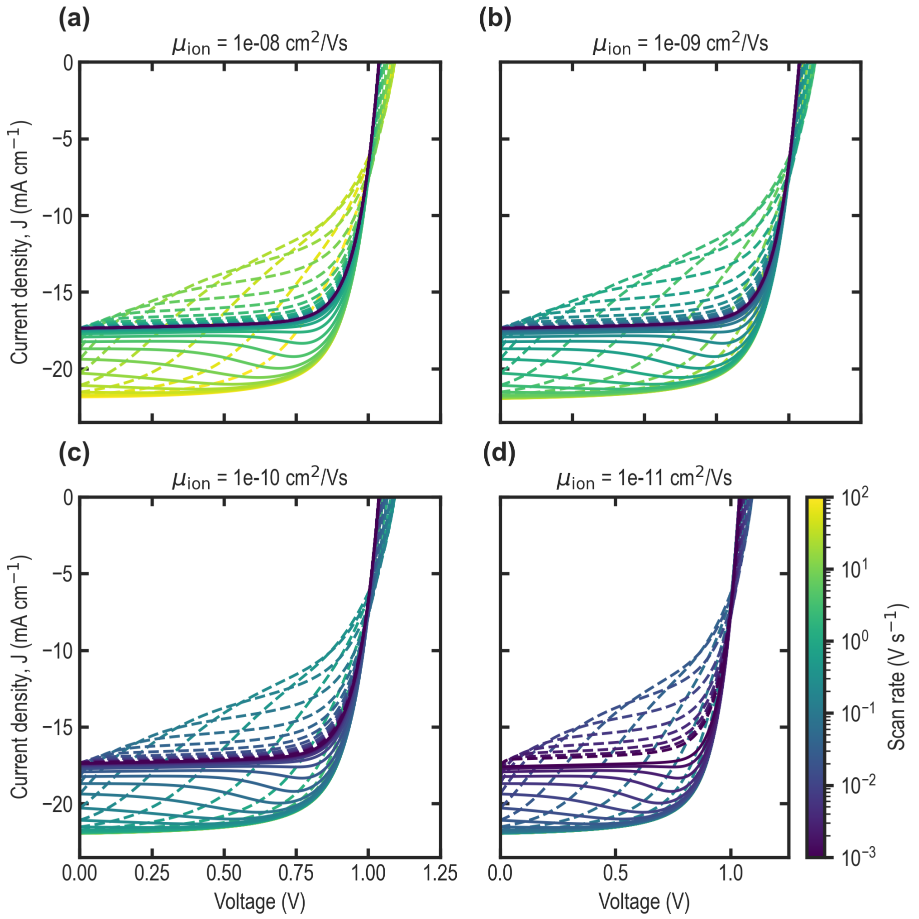

Figure S 10: (a)-(d) Simulated J-V curves for varying scan rates, with varying ion densities. The simulated transient starts from a steady-state precondition at illuminated open circuit, with the backward scan first (solid line), followed by the forward scan (dashed line), changing direction at short circuit. In this case $\Delta E_{\mathrm{CTL}} = 0.1$ eV, $V_{bi} = 1.0$ V.

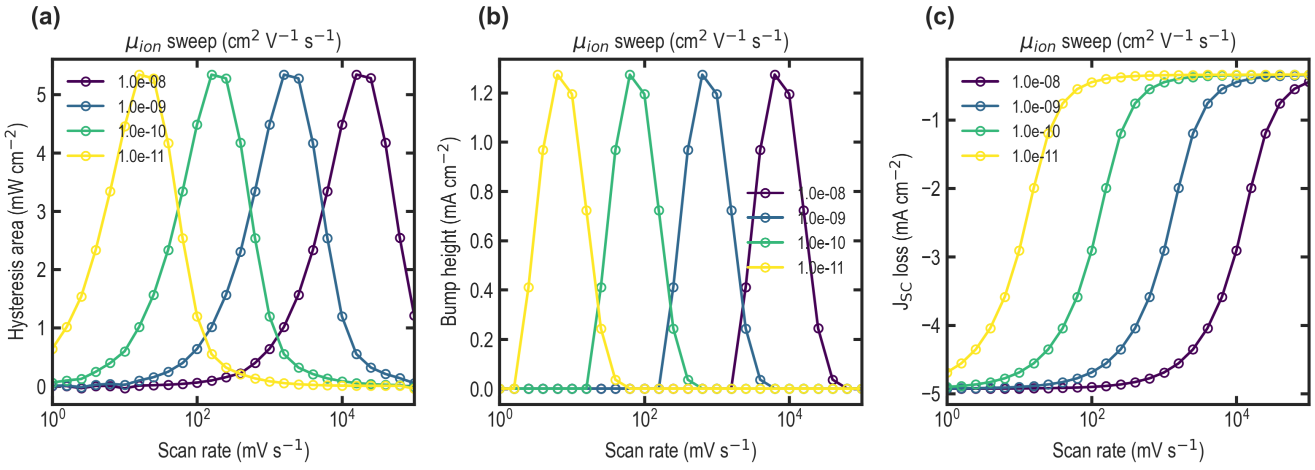

Figure S 11: (a) Hysteresis areas, (b) bump heights in the backward scan and (c) J_{SC} loss with respect to the maximum collectable photocurrent for the different scan rates, computed from the J-V curves in Fig. S10 for varying ion mobilities.

3.2.2 Evolution of ionic charge

Scan rate variation for a device with $\Delta E_{\mathbf{CTL}} = \mathbf{0.3}\,\mathbf{eV}$, $\mathbf{V_{bi}} = \mathbf{0.6}\,\mathbf{V}$:

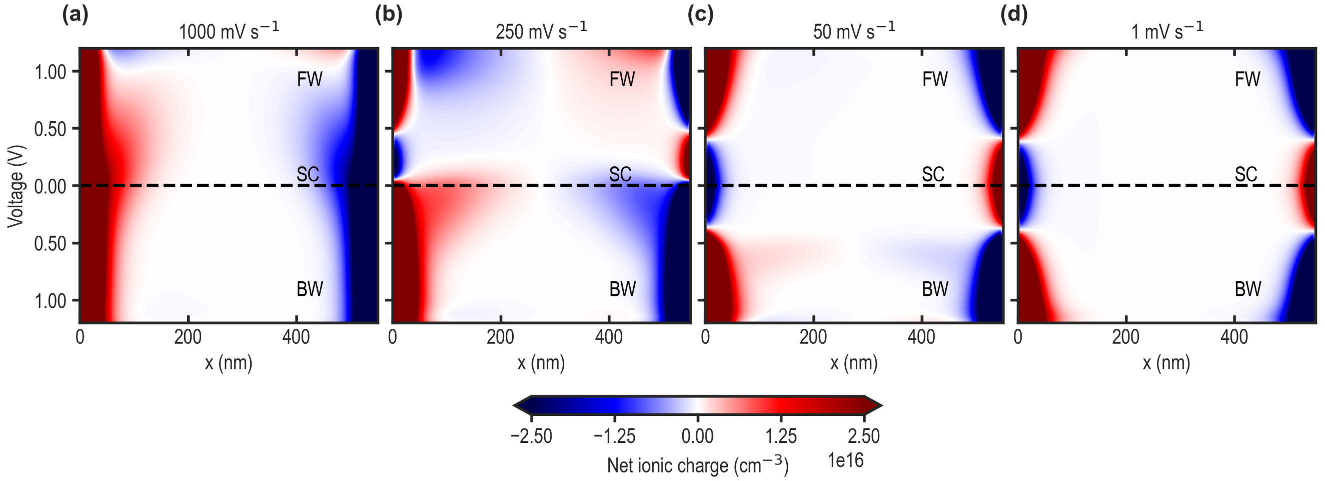

Figure S 12: (a)-(d) Net ionic charge profiles for the same simulations in Fig. 4 in the main text (J-V with varying scan rates). The voltage scan starts at 1.2 V in the BW direction after a steady state precalculation at V_{OC}, the maps evolve over time from bottom (BW scan) to short circuit (SC) to top (FW scan). The edges of the plots left and right at 0 nm and 550 nm correspond to the location of the interfaces with the ETL and HTL, respectively.

3.2.3 Evolution of quasi-Fermi level gradients

Scan rate variation for a device with $\Delta E_{\mathrm{CTL}} = 0.3\,\mathrm{eV}$, $\mathrm{V_{bi}} = 0.6\,\mathrm{V}$:

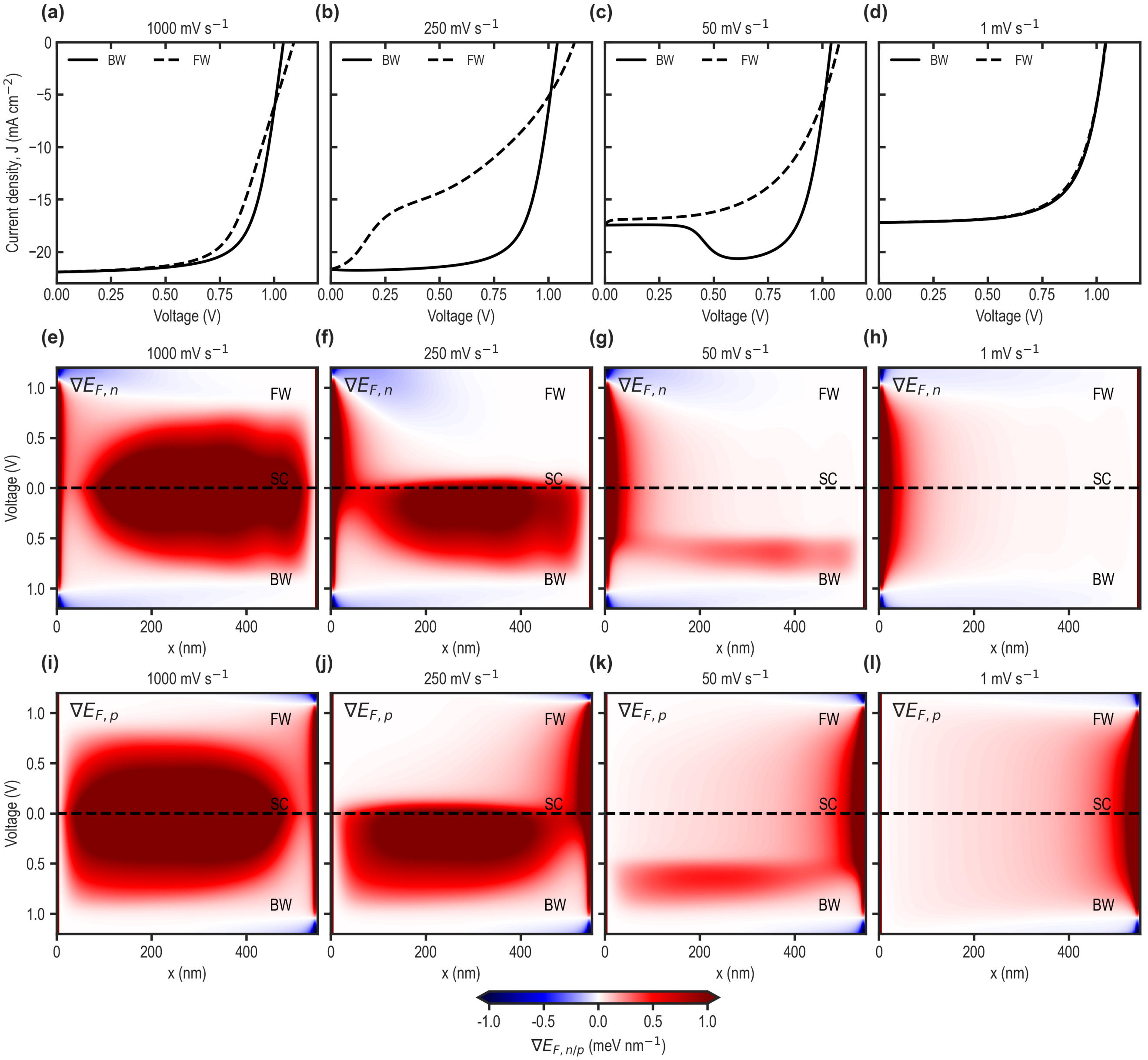

Figure S 13: (a)-(d) Simulated J-V curves with varying scan rates, starting with the BW scan from an illuminated $\mathrm{V_{OC}}$ precondition. Maps of the gradient of the quasi-Fermi levels (e)-(h) for electrons ($\nabla E_{F,n}$) and (i)-(l) for holes ($\nabla E_{F,p}$). The voltage scan starts at 1.2 V in the BW direction, the maps evolve over time from bottom (BW scan) to short circuit (SC) to top (FW scan). The edges of the plots left and right at 0 nm and 550 nm correspond to the location of the interfaces with the ETL and HTL, respectively.

3.2.4 Charge recombination mechanisms

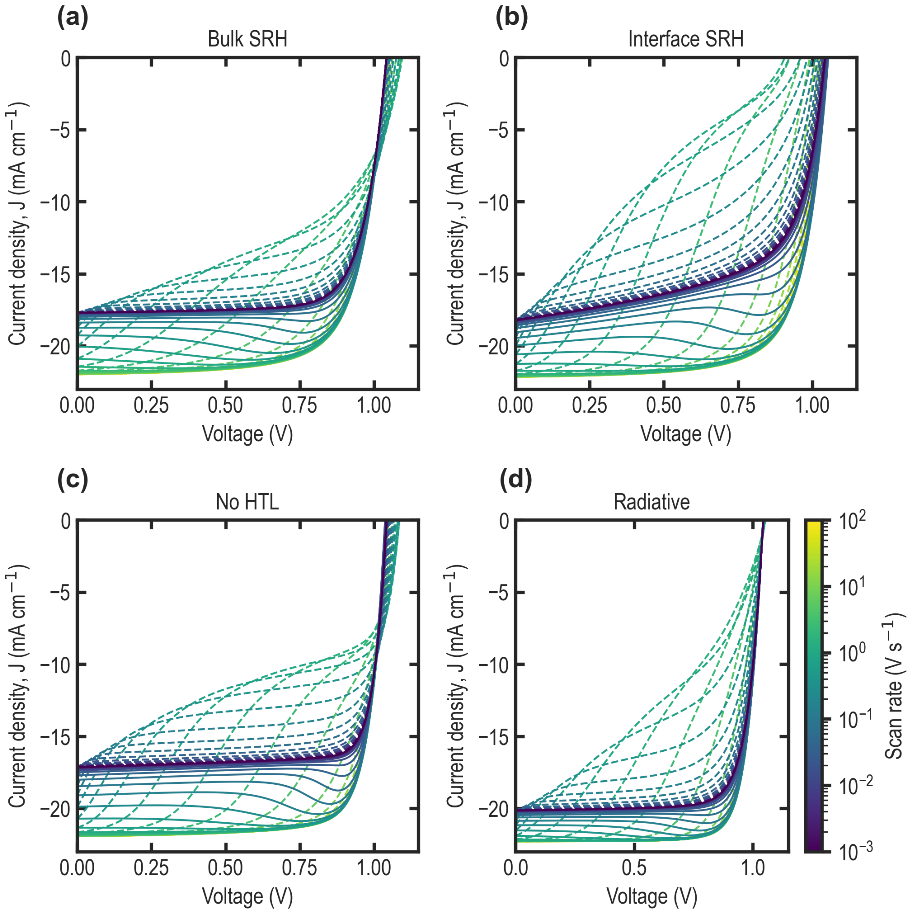

Figure S 14: Simulated J-V curves for different dominating recombination mechanisms, where recombination parameters have been adjusted to obtain the same steady-state V_{OC} values. The bump is present in all cases. (a) Dominating bulk SRH with a mid-gap defect state, with the carrier lifetime being $\tau_{n,p} = 39\,\mathrm{ns}$. (b) Dominating interface SRH at the ETL-perovskite surface, with velocity for electrons and holes $v_{\mathrm{int,SRH}} = 420\,\mathrm{cm\,s^{-1}}$, at an energy level half-way between the perovskite VB and ETL CB levels. (c) Non-selective back-contact, where the HTL has been removed altogether. (d) Only radiative recombination considered, with high bimolecular recombination, $\beta_\gamma = 6\times10^{-8}\,\mathrm{cm^3\,s^{-1}}$.

Band-to-band recombination only, with $\beta_\gamma = 1 \times 10^{-10}\,\mathrm{cm^3\,s^{-1}}$ and varying electronic mobility in perovskite:

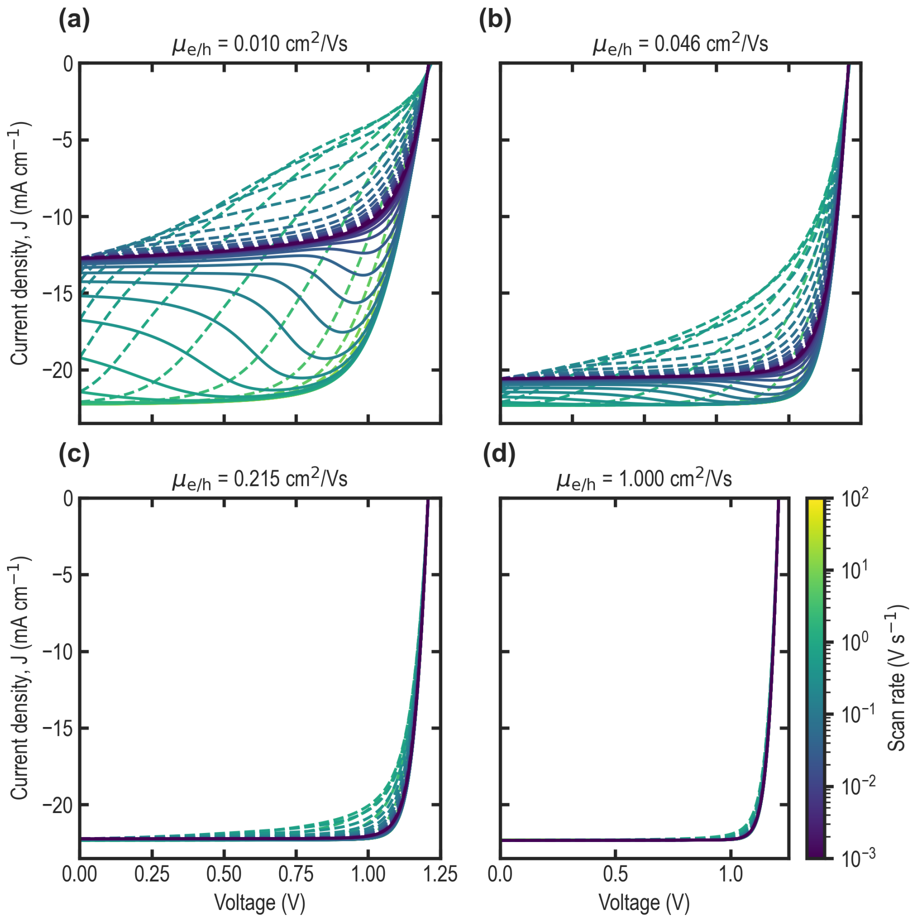

Figure S 15: (a)-(d) Simulated J-V curves for varying scan rates, with varying mobility levels for electrons and holes in perovskite, showing how transport-dominated losses can also manifest as a substantial J_{SC} loss and bump, without varying ionic parameters.

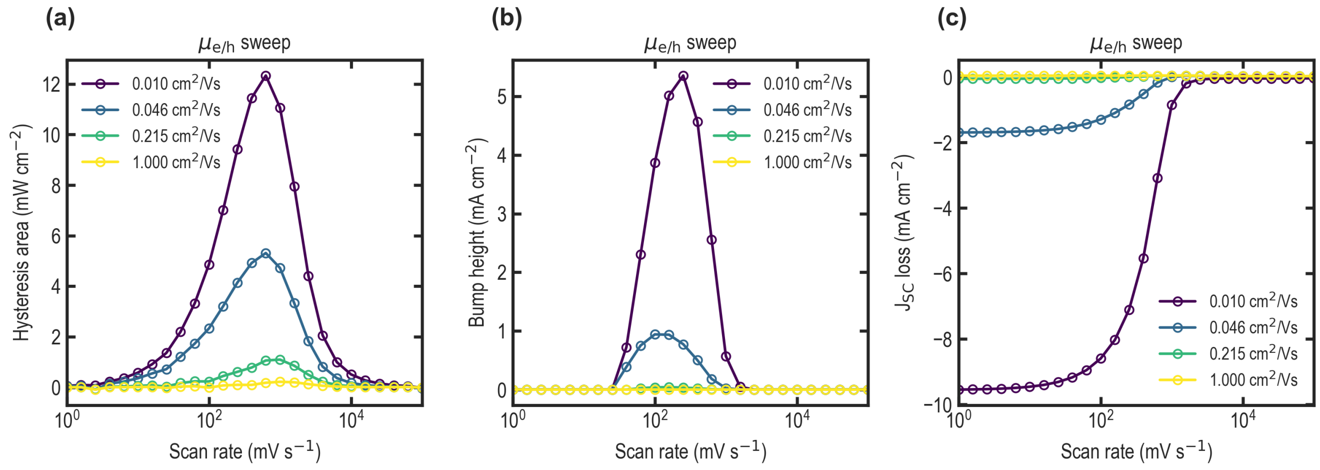

Figure S 16: (a) Hysteresis areas, (b) bump heights in the backward scan and (c) J_{SC} loss with respect to the maximum collectable photocurrent for the different scan rates, computed from the J-V curves in Fig. S15.

3.2.5 Energy level sweeps

The parameter ΔE_{CTL} is defined as the energy difference between the CB of the PSK and the CB of the ETL (downwards in energy), as well as between the VB of the PSK and the VB of the HTL (upwards). In a first sweep, the WF of the electrodes is varied in parallel to keep the electrode charge constant, which also modifies V_{bi} during the sweep. This is then compared to the effect of varying the CTL levels alone, whilst keeping the WF of the electrodes (thus V_{bi}) constant at different levels.

Varying ΔE_{CTL} and V_{bi} in parallel (constant electrode charge density):

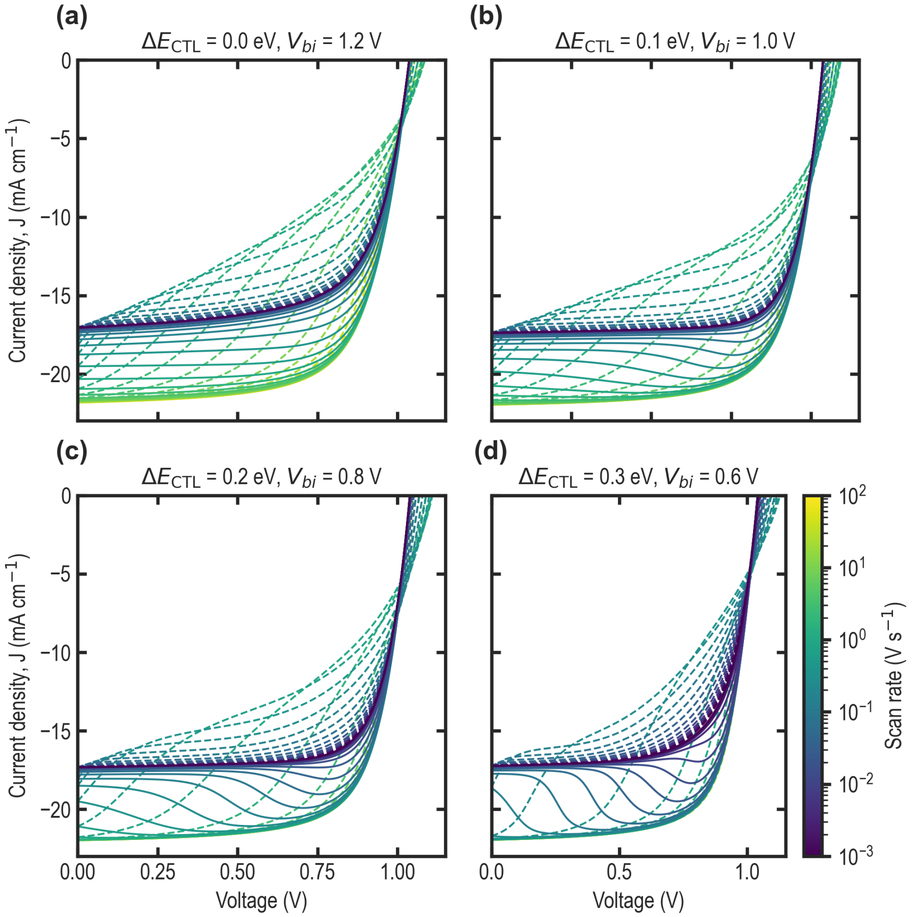

Figure S 17: (a)-(d) Simulated J-V curves for varying scan rates, with varying ΔE_{CTL} and V_{bi} levels (with constant electrode charge density). The simulated transient starts from a steady-state precondition at illuminated open circuit, with the backward scan first (solid line), followed by the forward scan (dashed line), changing direction at short circuit.

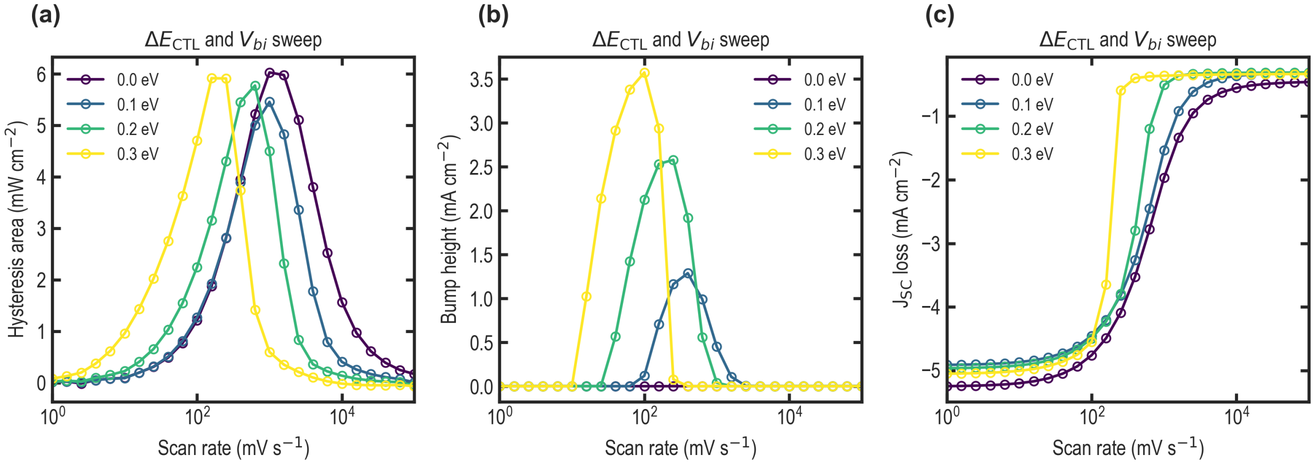

Figure S 18: (a) Hysteresis areas, (b) bump heights in the backward scan and (c) J_{SC} loss with respect to the maximum collectable photocurrent for the different scan rates, computed from the J-V curves in Fig. S17 for varying ΔE_{CTL} and V_{bi} levels (with constant electrode charge density). For perfectly aligned contacts ($\Delta E_{\mathrm{CTL}} = 0\,\mathrm{eV}$) there is no bump in the backward scan.

Steady-state ionic charge maps at different voltages, for varying ΔE_{CTL} and V_{bi}:

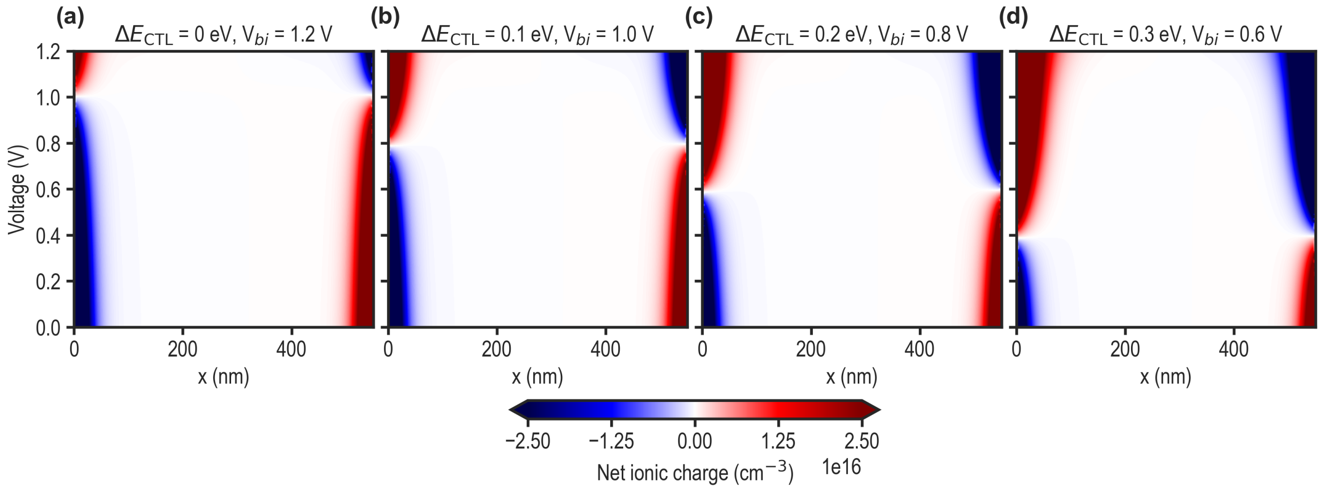

Figure S 19: (a)-(d) Net ionic charge profiles at different voltages at steady-state, for varying the energy levels of the contacts (ΔE_{CTL} and V_{bi} in parallel). The bump becomes more likely the lower the 'ion-free' voltage level.

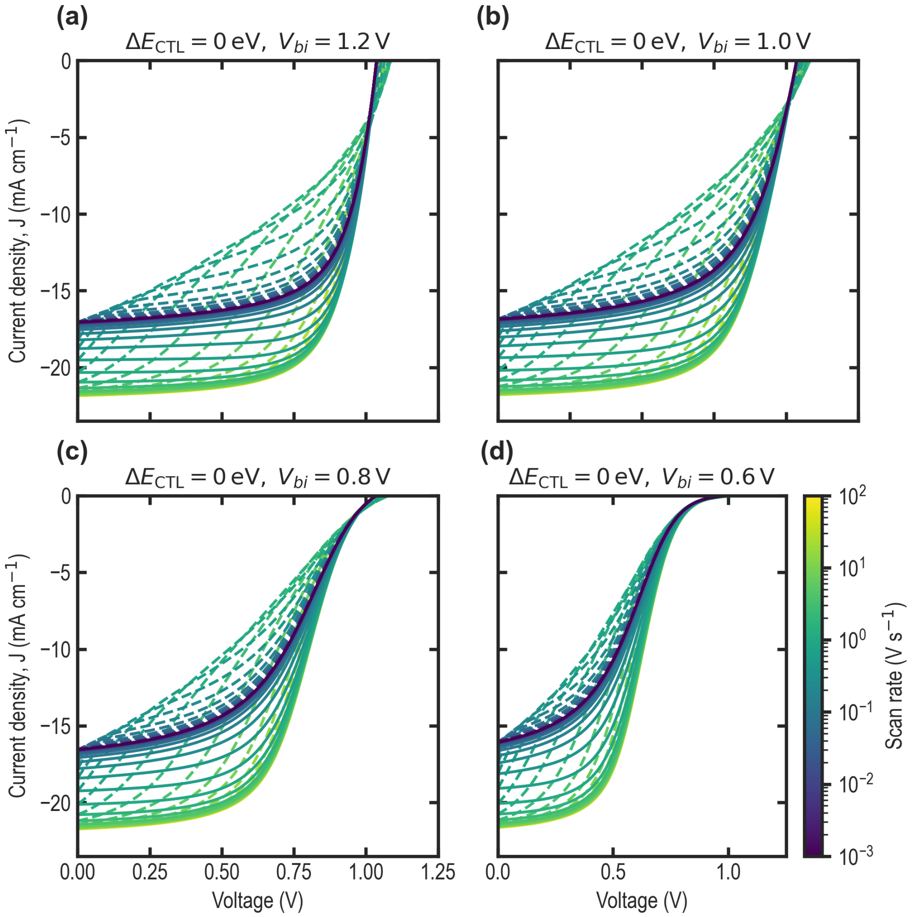

Figure S 20: (a)-(d) Simulated J-V curves for varying scan rates, for varying V_{bi} levels with aligned CTLs, $\Delta E_{\mathrm{CTL}} = 0\,\mathrm{eV}$. The simulated transient starts from a steady-state precondition at illuminated open circuit, with the backward scan first (solid line), followed by the forward scan (dashed line), changing direction at short circuit.

Steady-state ionic charge maps at different voltages, for varying $\mathbf{V_{bi}}$ with $\Delta E_{\mathbf{CTL}} = 0\,\mathbf{eV}$:

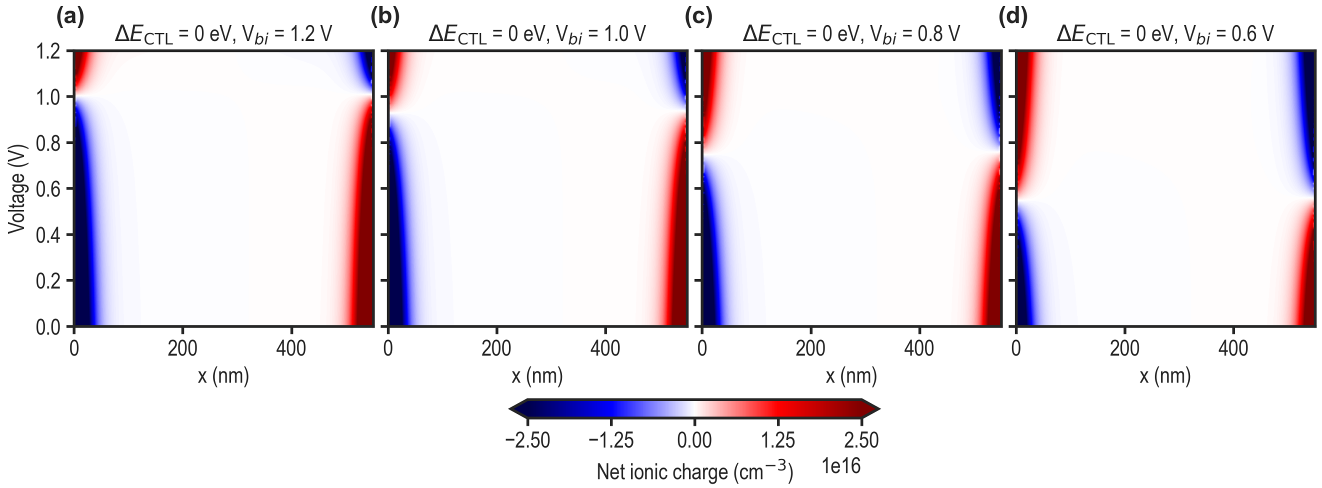

Figure S 21: (a)-(d) Net ionic charge profiles at different voltages at steady-state, for varying V_{bi} alone.

Transient ionic charge at short-circuit from V_{OC} precondition, for varying ΔE_{CTL} and V_{bi}:

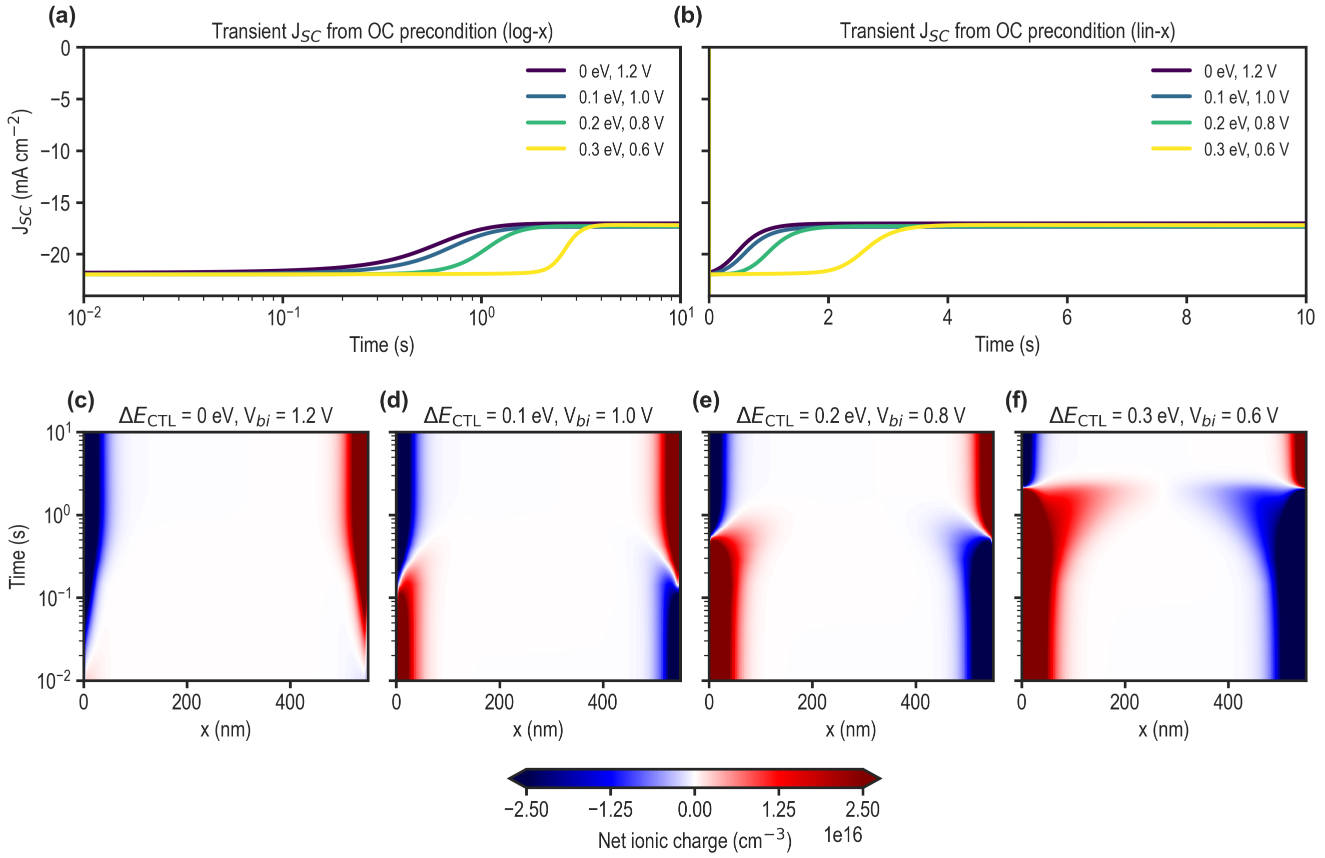

Figure S 22: (a)-(b) Transient J_{SC} upon a change from illuminated open-circuit conditions and (c)-(f) corresponding transient net ionic charge profiles, for varying the energy levels of the contacts (ΔE_{CTL} and V_{bi} in parallel).

Perfectly aligned contacts ($\Delta E_{CTL} = 0\,eV$, $V_{bi} = 1.2\,V$), showing no bump:

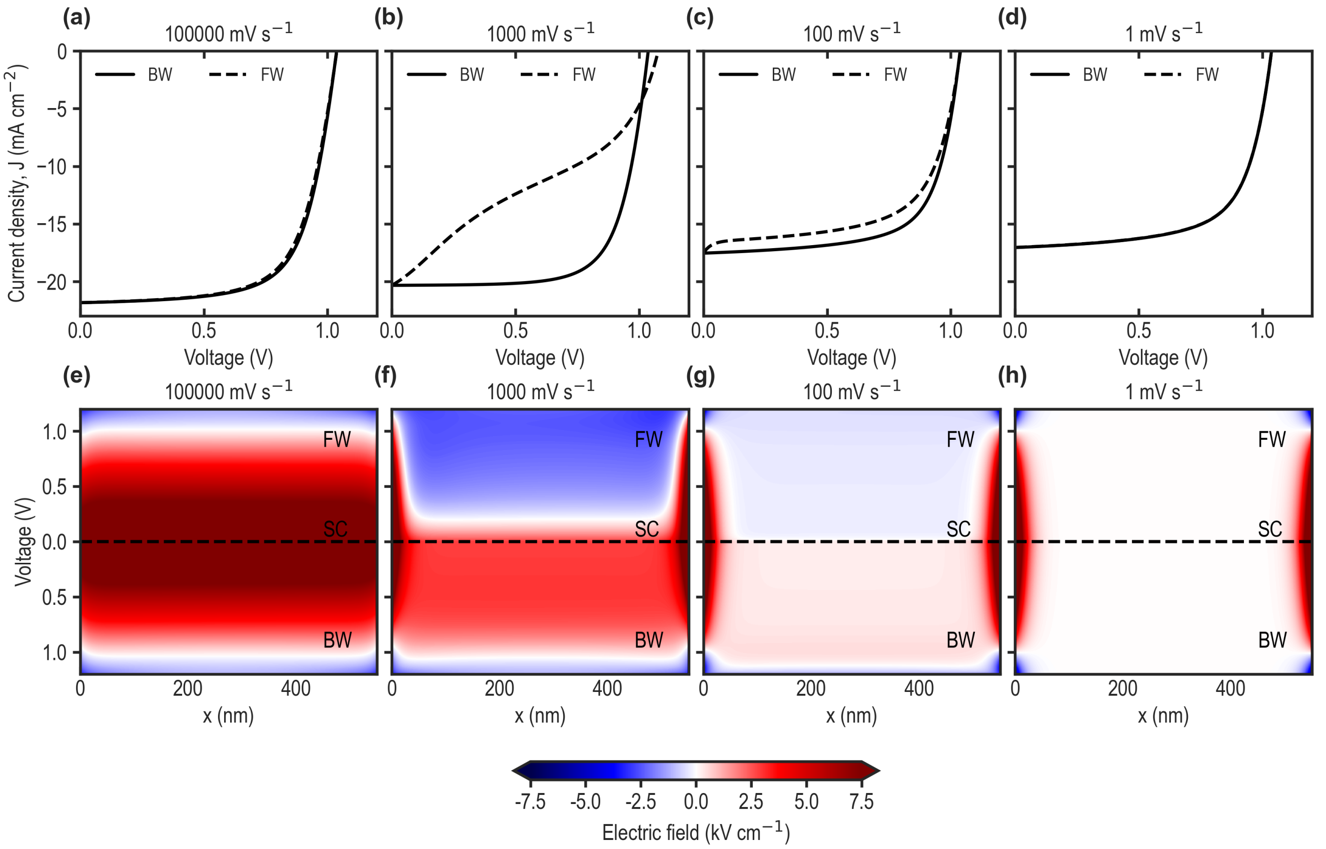

Figure S 23: (a)-(d) Simulated J-V curves with varying scan rates for perfectly aligned contacts ($\Delta E_{CTL} = 0\,eV$), starting with the BW scan from an illuminated V_{OC} precondition. (e)-(h) Electric field profiles in perovskite during the J-V scans above, for different scan rates. The voltage scan starts at 1.2 V in the BW direction, so the electric field evolves over time from bottom (BW scan) to short circuit (SC) to top (FW scan). The edges of the plots at 0 nm and 550 nm correspond to the location of the interfaces with the ETL and HTL, respectively.

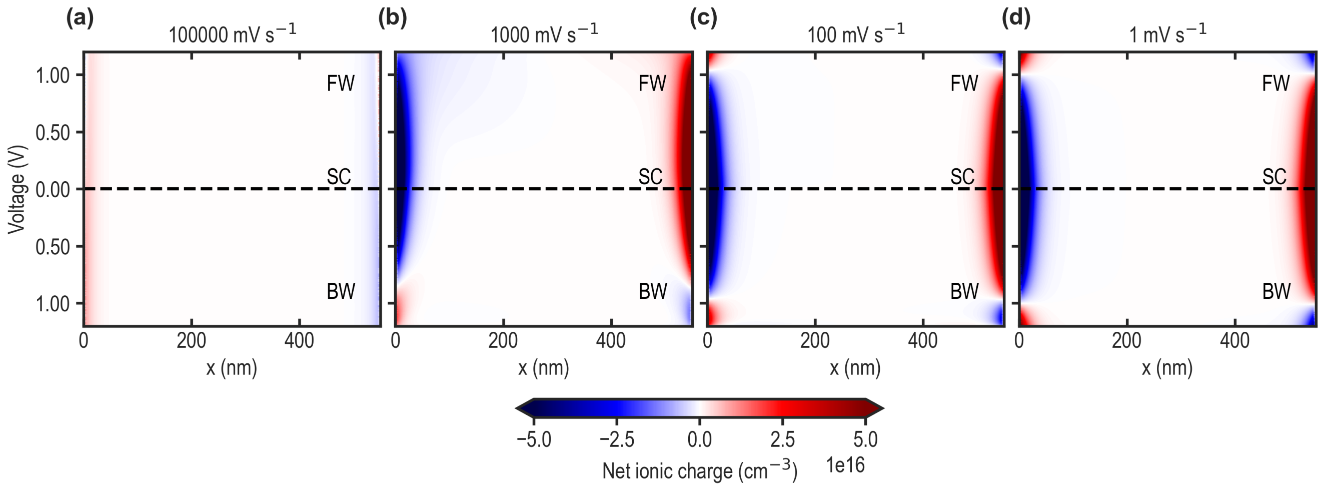

Figure S 24: (a)-(d) Net ionic charge profiles for the same simulations above (J-V with varying scan rates), for perfectly aligned contacts ($\Delta E_{CTL} = 0\,eV$, $V_{bi} = 1.2\,V$).

Varying $\Delta E_{\mathbf{CTL}}$ with constant electrode WF level ($\mathbf{V_{bi} = 1\,V}$):

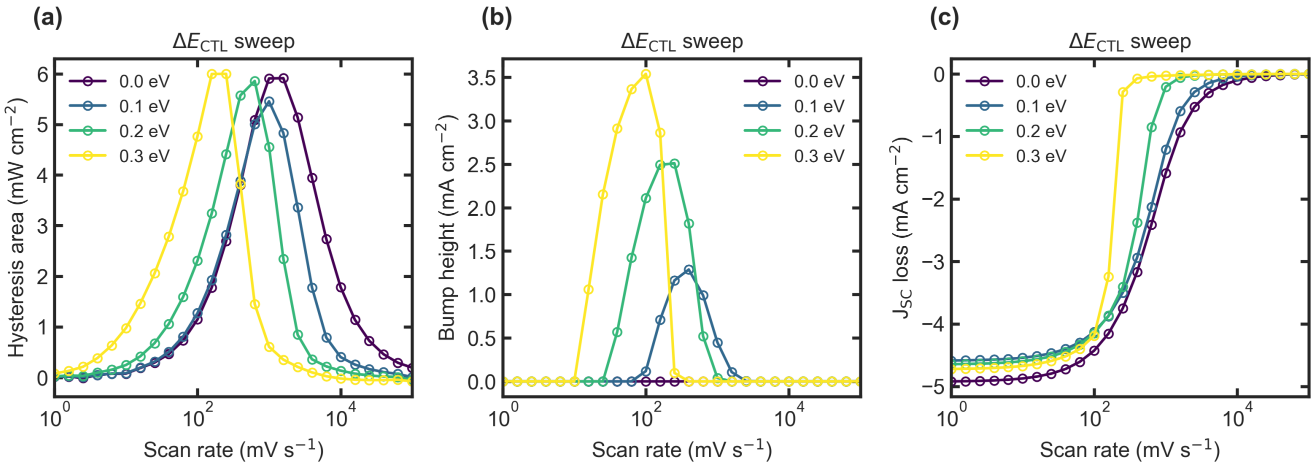

Figure S 25: (a) Hysteresis areas, (b) bump heights in the backward scan and (c) J_{SC} loss with respect to the maximum collectable photocurrent for the different scan rates, for varying ΔE_{CTL} whilst keeping $V_{bi} = 1\,V$ constant. The results are identical as in Fig. S18, highlighting the importance of the energetic offset with the CTLs.

HTL-free PSC with dominating current losses at the non-selective back-contact:

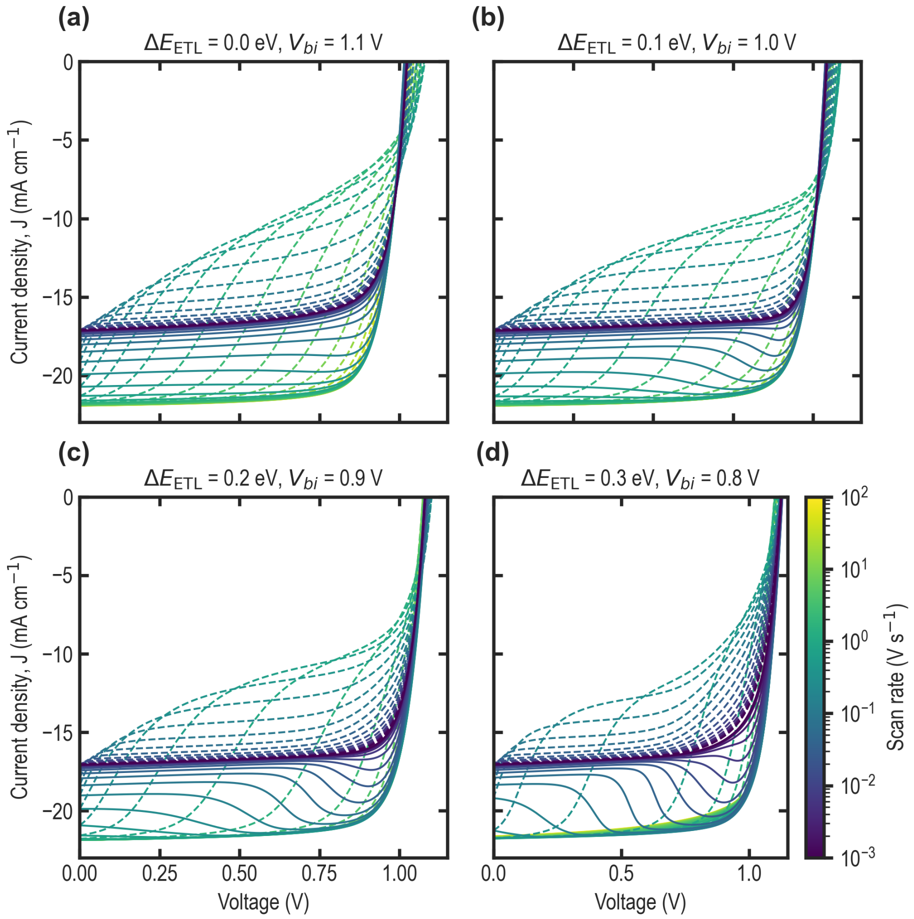

Figure S 26: Simulated J-V curves for various scan rates for dominating recombination at the non-selective back-contact (no bulk SRH considered), where the HTL has been removed altogether and the back-electrode WF is kept constant at 5.3 eV. (a)-(d) show different ΔE_{ETL} and V_{bi} levels, by modifying the energetic alignment at the front-contact only.

3.2.6 CTL permittivity

Varying $\epsilon_{\mathbf{CTL}}$ with constant $\Delta E_{\mathbf{CTL}} = 0.1\,\mathrm{eV}$:

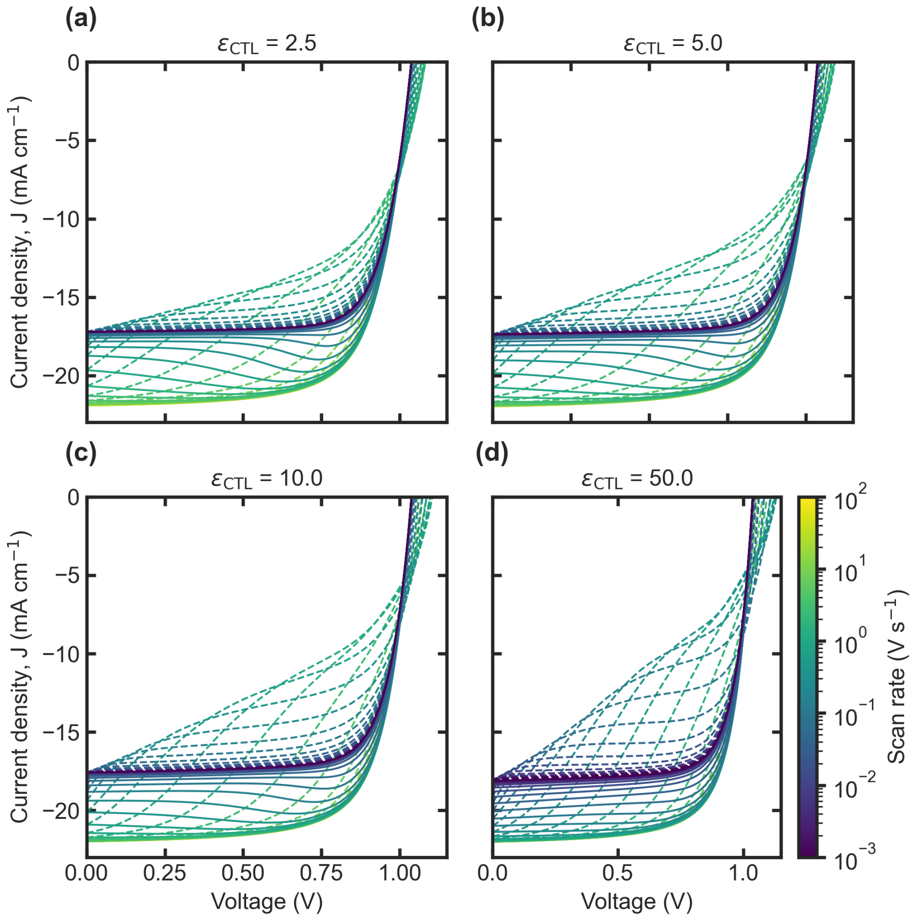

Figure S 27: (a)-(d) Simulated J-V curves for varying scan rates, with varying ϵ_{CTL}.

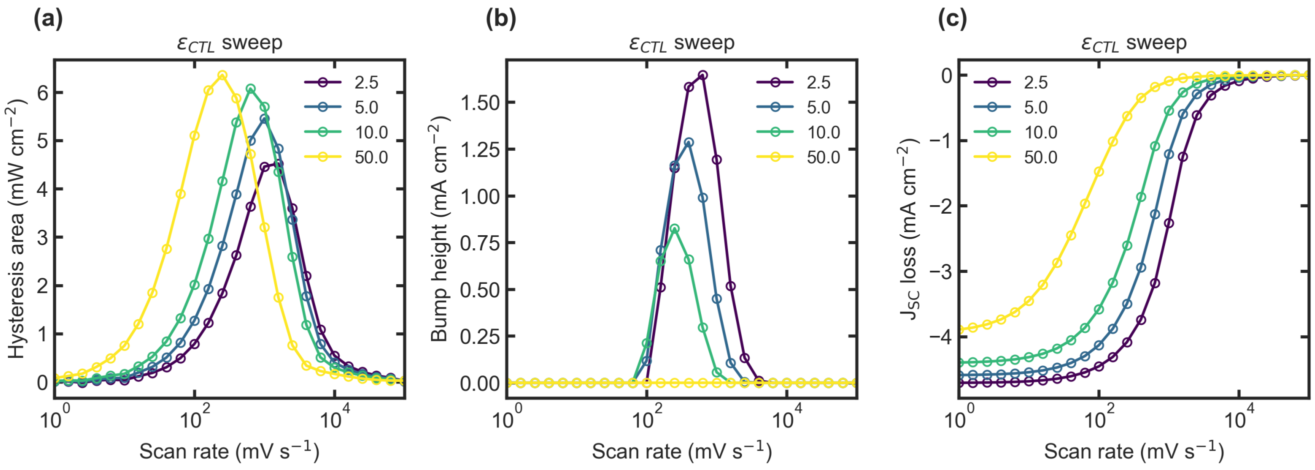

Figure S 28: (a) Hysteresis areas, (b) bump heights in the backward scan and (c) J_{SC} loss with respect to the maximum collectable photocurrent for the different scan rates, computed from the J-V curves in Fig. S27 for varying ϵ_{CTL}.

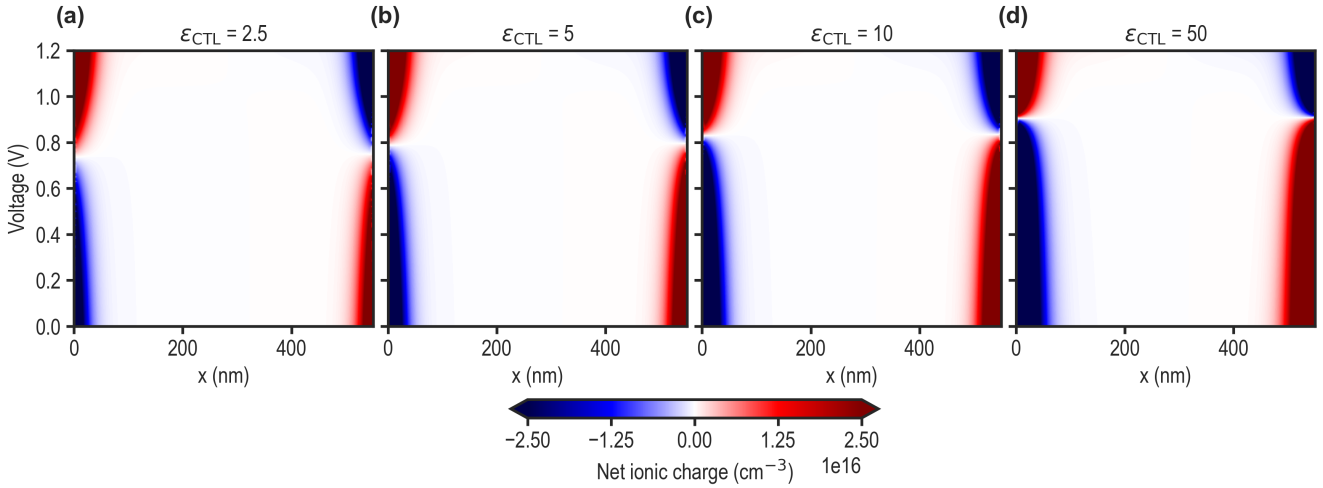

Figure S 29: (a)-(d) Net ionic charge profiles at different voltages at steady-state, for varying ϵ_{CTL}. The 'ion-free' voltage level increases with increasing ϵ_{CTL}.

Low CTL permittivity ($\epsilon_{CTL} = 2.5$):

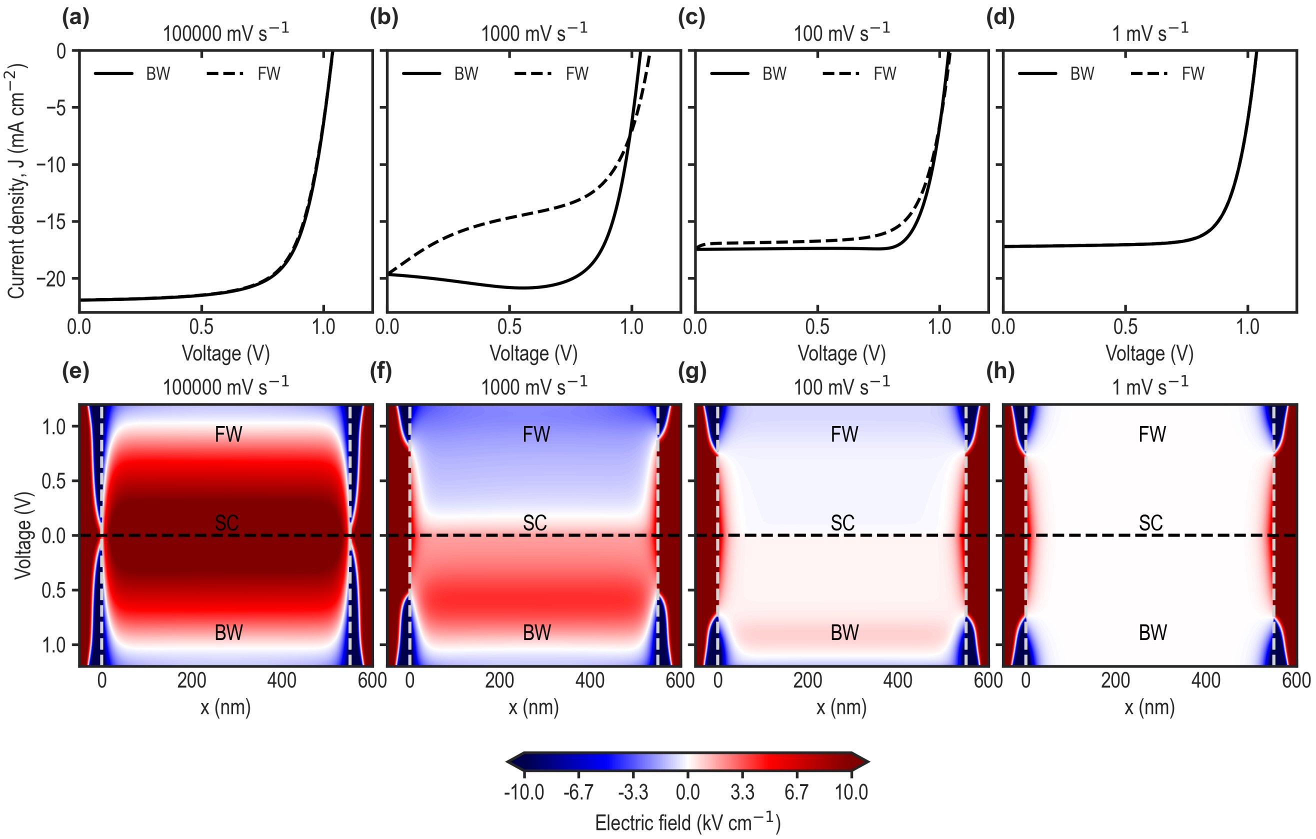

Figure S 30: (a)-(d) Simulated J-V curves with varying scan rates for low CTL permittivity ($\epsilon_{CTL} = 2.5$), starting with the BW scan from an illuminated V_{OC} precondition. (e)-(h) Electric field profiles in perovskite and CTLs during the J-V scans above, for different scan rates. The voltage scan starts at 1.2 V in the BW direction, so the electric field evolves over time from bottom (BW scan) to short circuit (SC) to top (FW scan). The locations at 0 nm an 550 nm corresponds to the ETL- and HTL-perovskite interfaces, respectively.

High CTL permittivity ($\epsilon_{CTL} = 50$):

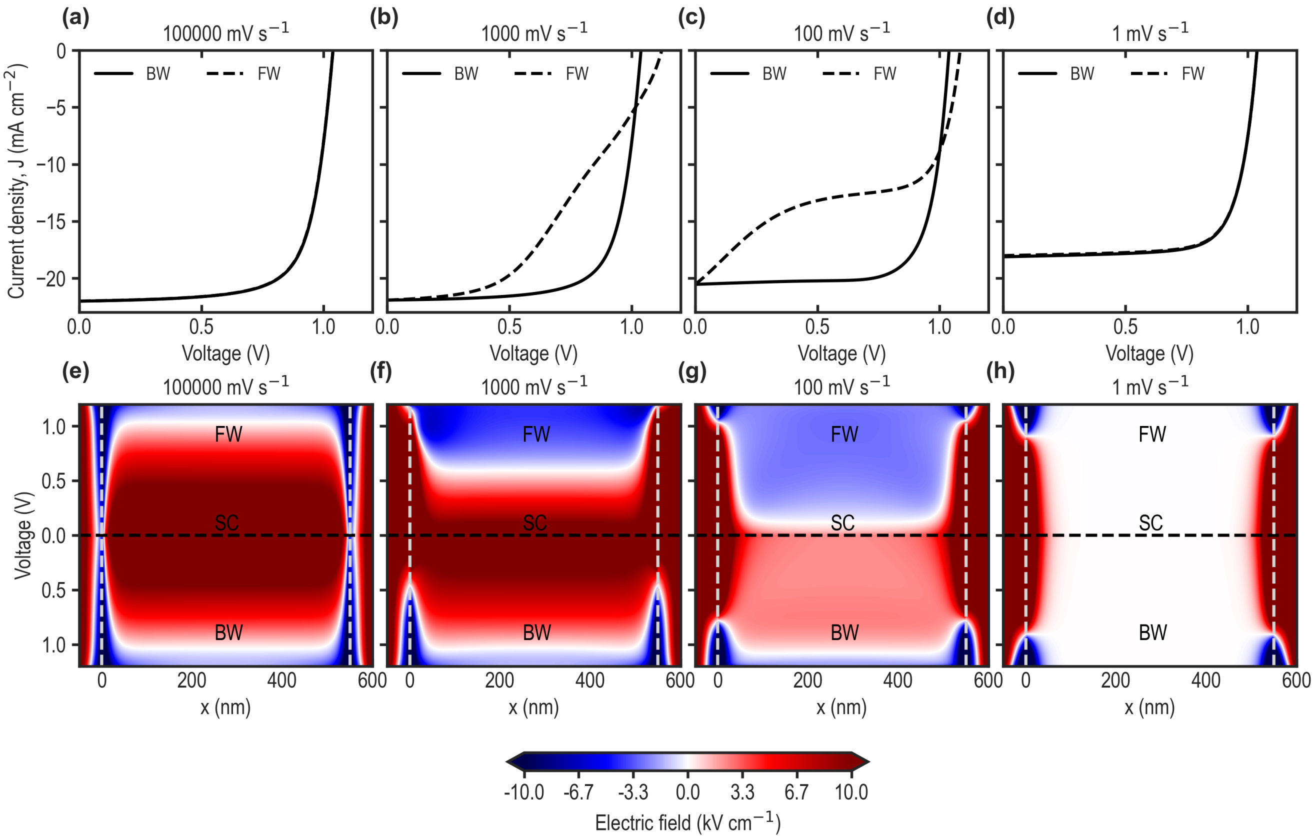

Figure S 31: (a)-(d) Simulated J-V curves with varying scan rates for high CTL permittivity ($\epsilon_{CTL} = 50$), starting with the BW scan from an illuminated V_{OC} precondition. (e)-(h) Electric field profiles in perovskite and CTLs during the J-V scans above, for different scan rates. The voltage scan starts at 1.2 V in the BW direction, so the electric field evolves over time from bottom (BW scan) to short circuit (SC) to top (FW scan). The locations at 0 nm an 550 nm corresponds to the ETL- and HTL-perovskite interfaces, respectively.

3.2.7 Comparison with and without ions

Steady-state electric field profiles at V_{OC}:

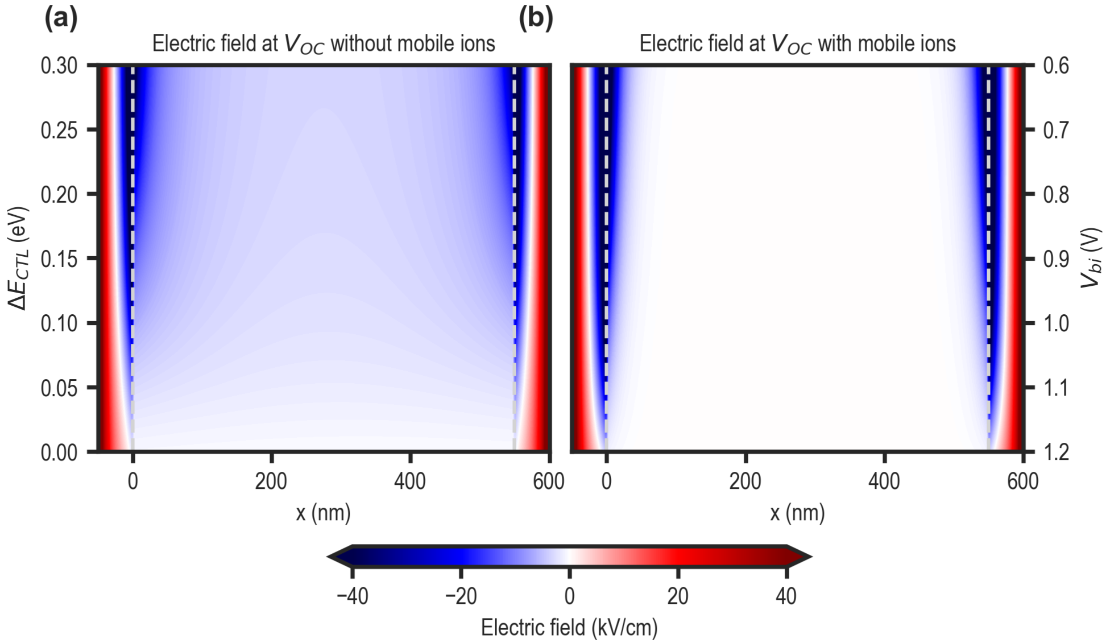

Figure S 32: Steady-state electric field profiles along the depth of the solar cell for equivalent devices (a) without mobile ions and (b) with mobile ions, with varying ΔE_{CTL} and V_{bi} in parallel.

Steady-state J-V curves with and without mobile ions for the parameter sets in Table 1, varying the energetic alignment (ΔE_{CTL} and V_{bi} in parallel):

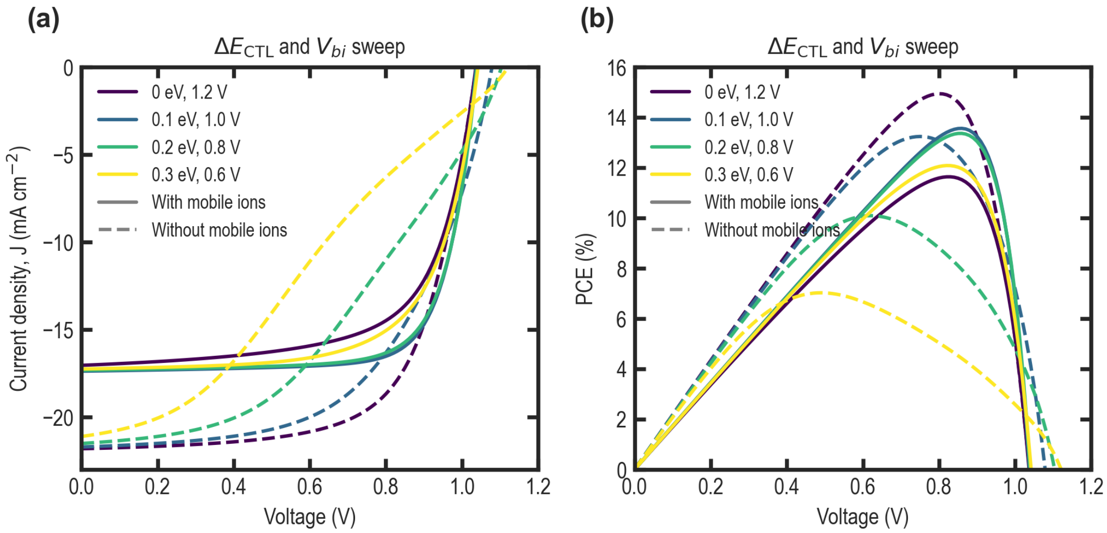

Figure S 33: (a) Steady-state J-V curves and (b) PCE-V for equivalent devices with varying energetic offsets, with and without mobile ions.

Steady-state J-V curves with and without mobile ions for a device without HTL, varying the energetic alignment of the front-contact only ($\Delta E_{\mathbf{ETL}}$ and $\mathbf{V_{bi}}$ in parallel):

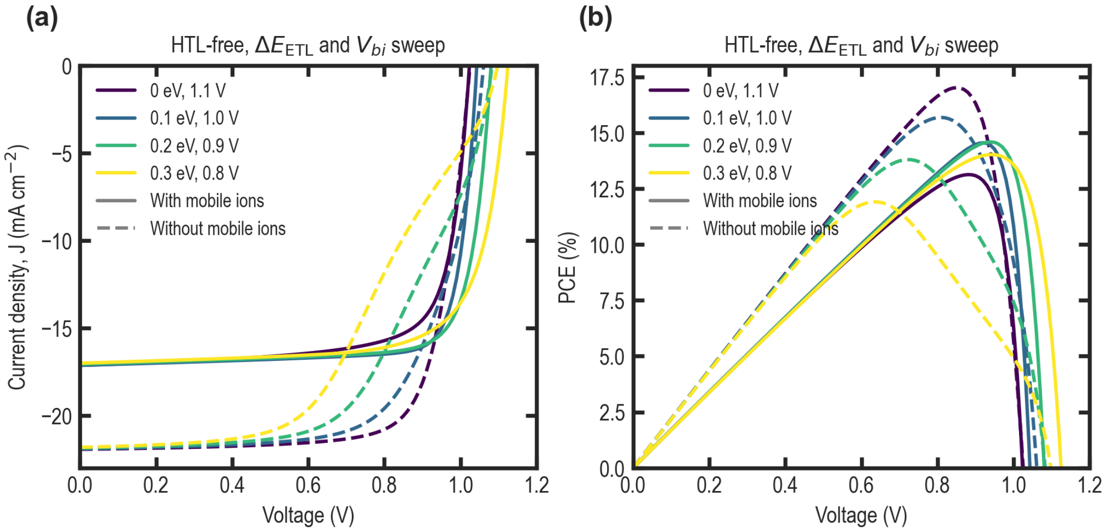

Figure S 34: (a) Steady-state J-V curves and (b) PCE-V for equivalent devices with varying energetic offsets, with and without mobile ions, for a HTL-free situation as in Fig S21. The back-electrode WF is kept constant at 5.3 eV.

References

(1) D. Bogachuk, R. Tsuji, D. Martineau, S. Narbey, J. P. Herterich, L. Wagner, K. Suginuma, S. Ito and A. Hinsch, *Carbon*, 2021, **178**, 10–18.

(2) S. G. Hashmi, D. Martineau, M. I. Dar, T. T. Myllymäki, T. Sarikka, V. Ulla, S. M. Zakeeruddin and M. Grätzel, *Journal of Materials Chemistry A*, 2017, **5**, 12060–12067.

(3) *Fluxim AG, SETFOS Simulation Software for Organic and Perovskite Solar Cells and LEDs*, https://www.fluxim.com/setfos-intro (visited on 02/28/2024).

(4) S. Sarkar, V. Gupta, M. Kumar, J. Schubert, P. T. Probst, J. Joseph and T. A. König, *ACS applied materials & interfaces*, 2019, **11**, 13752–13760.
